# Supplementary figures and images for: Organotypic culture of human brain explants as a preclinical model for AI-driven antiviral studies
Source: EMBO Mol Med. 2024 Mar 12;16(4):1004–26. doi: 10.1038/s44321-024-00039-9 (PMC11018746; doi:10.1038/s44321-024-00039-9)

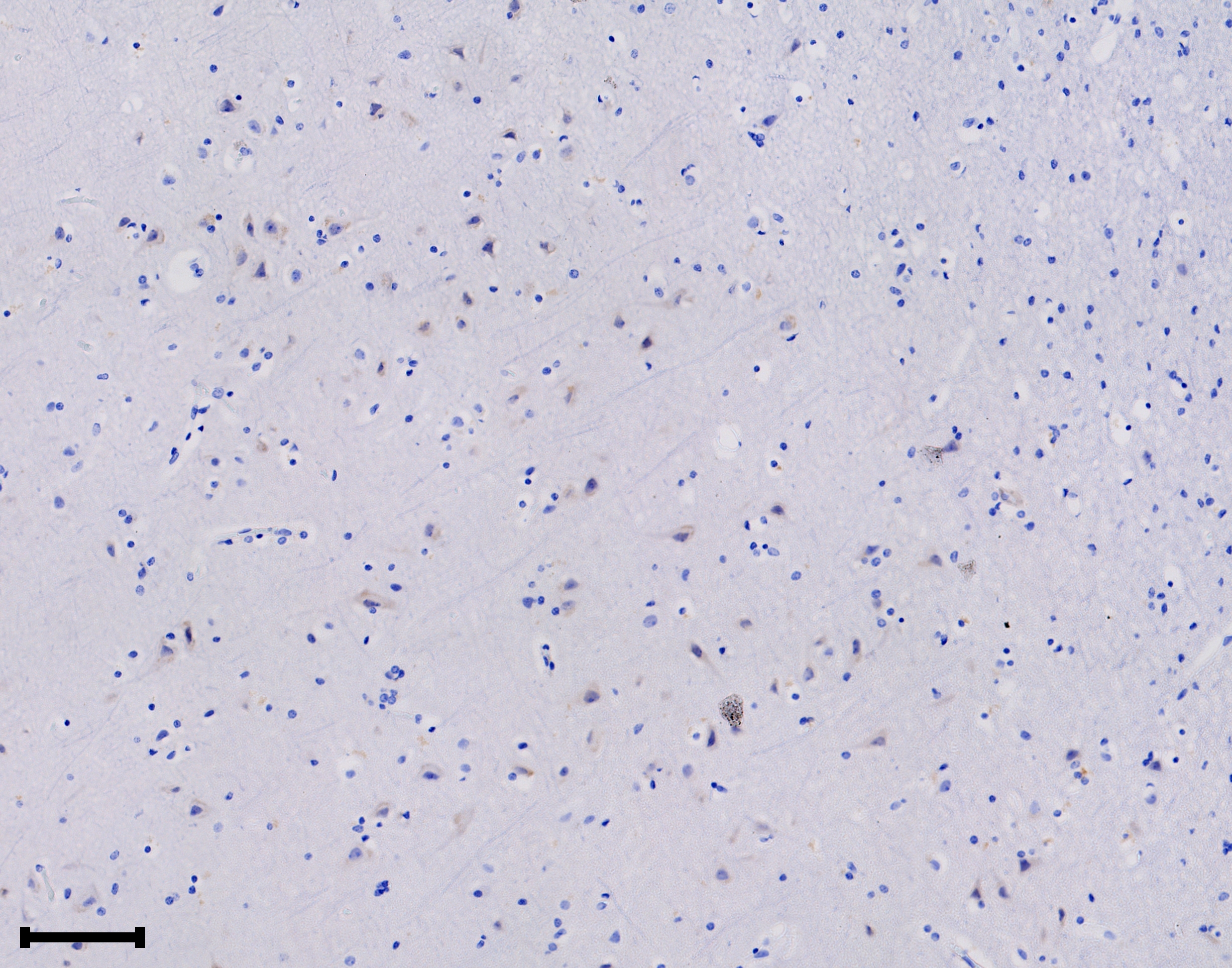

Supplement: Supplementary file 3 — Source Data Fig. 1 [file 44321_2024_39_MOESM3_ESM.zip › Figure 1/C/Parietal J0 MERGE_TileScan 2 Merged_Crop001_ch00_SV.jpg]

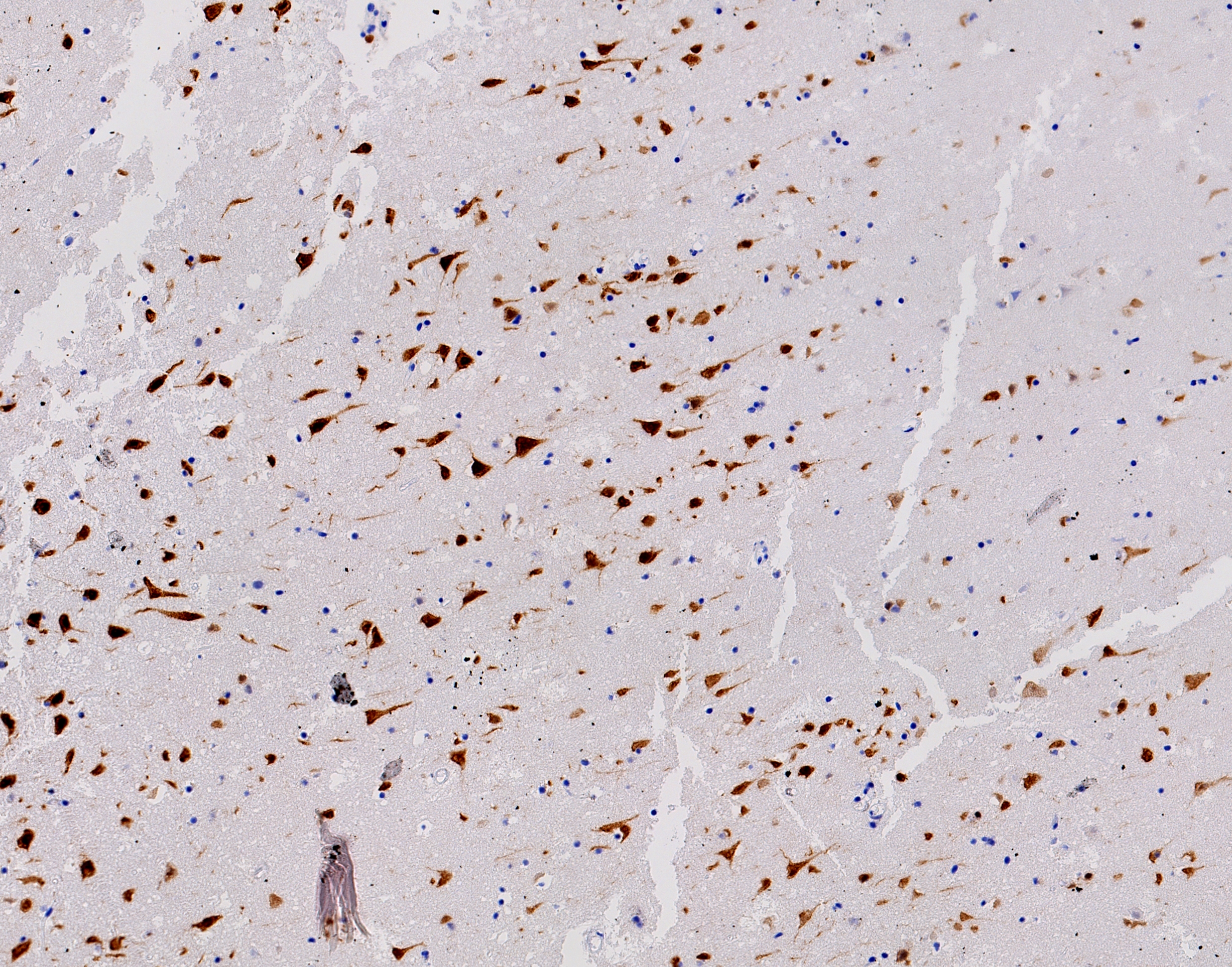

Supplement: Supplementary file 3 — Source Data Fig. 1 [file 44321_2024_39_MOESM3_ESM.zip › Figure 1/C/Parietal J12 MERGE_TileScan 2 Merged_Crop001_ch00_SV.jpg]

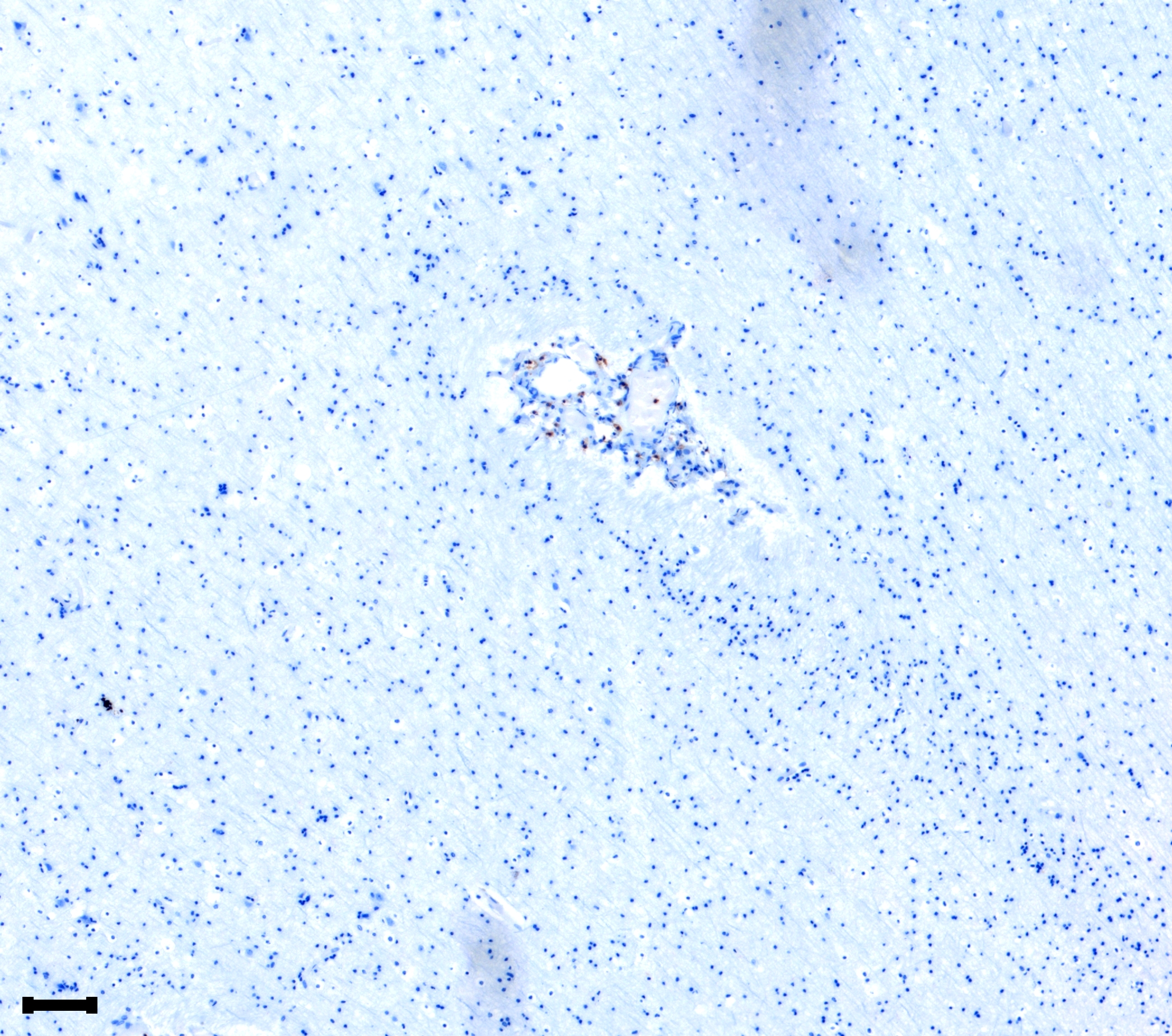

Supplement: Supplementary file 3 — Source Data Fig. 1 [file 44321_2024_39_MOESM3_ESM.zip › Figure 1/D/Project_A1 Region1_Merged_Crop001_ch00_SV.jpg]

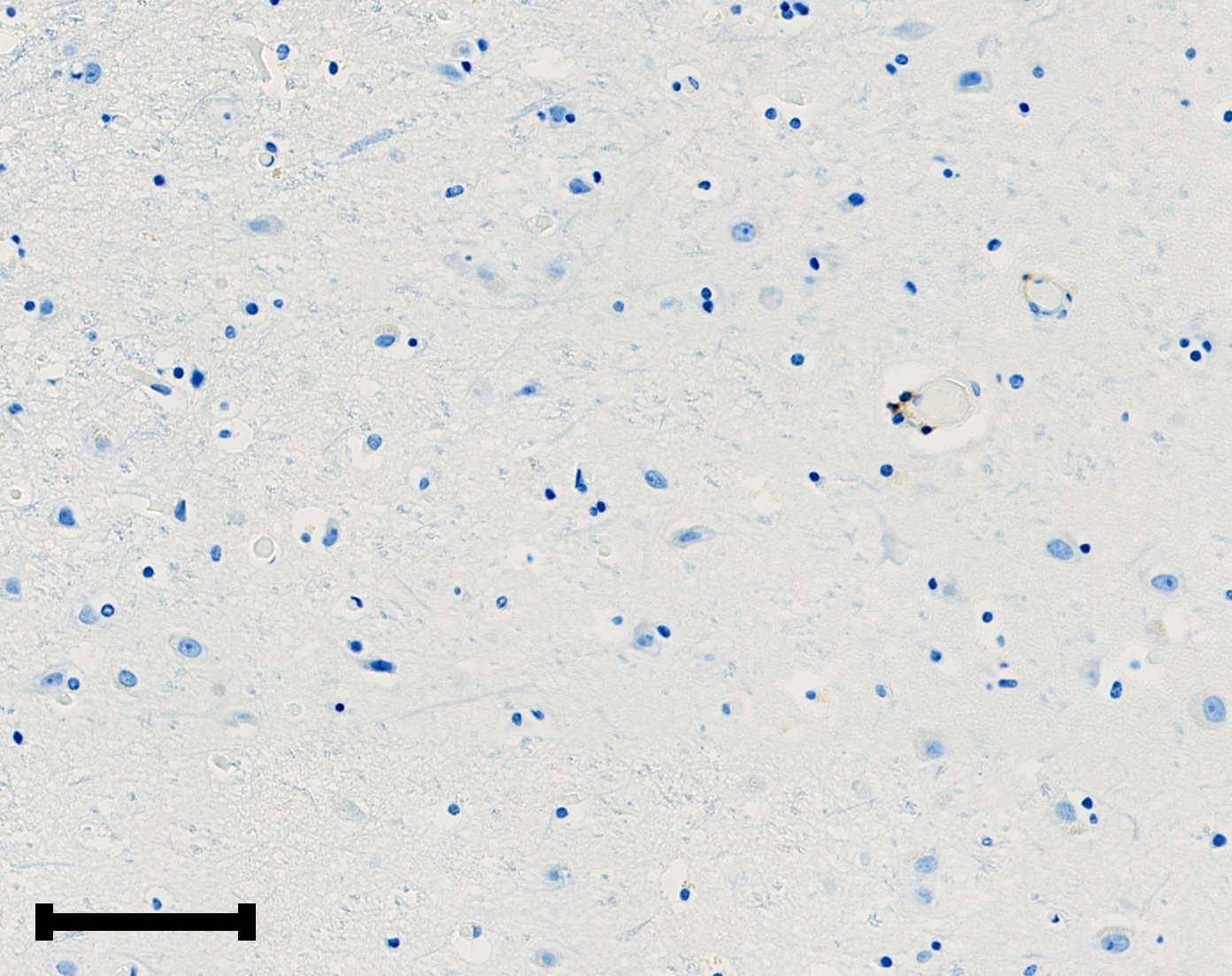

Supplement: Supplementary file 3 — Source Data Fig. 1 [file 44321_2024_39_MOESM3_ESM.zip › Figure 1/E/53 merge_TileScan 2 Merged_Crop001_Crop002_ch00_SV.jpg]

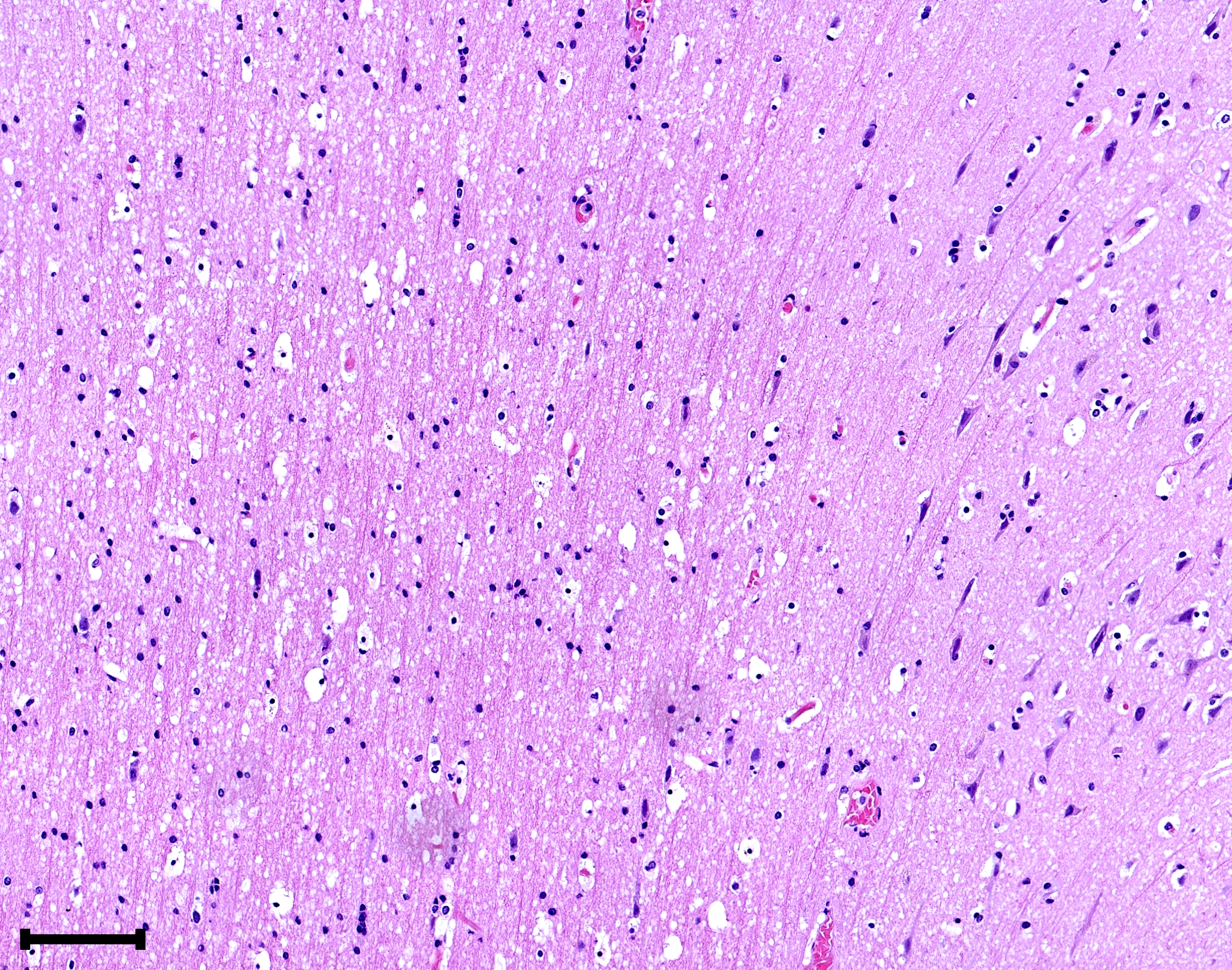

Supplement: Supplementary file 3 — Source Data Fig. 1 [file 44321_2024_39_MOESM3_ESM.zip › Figure 1/B/Parietal J0 MERGE_TileScan 1 Merged_Crop001_ch00_SV.jpg]

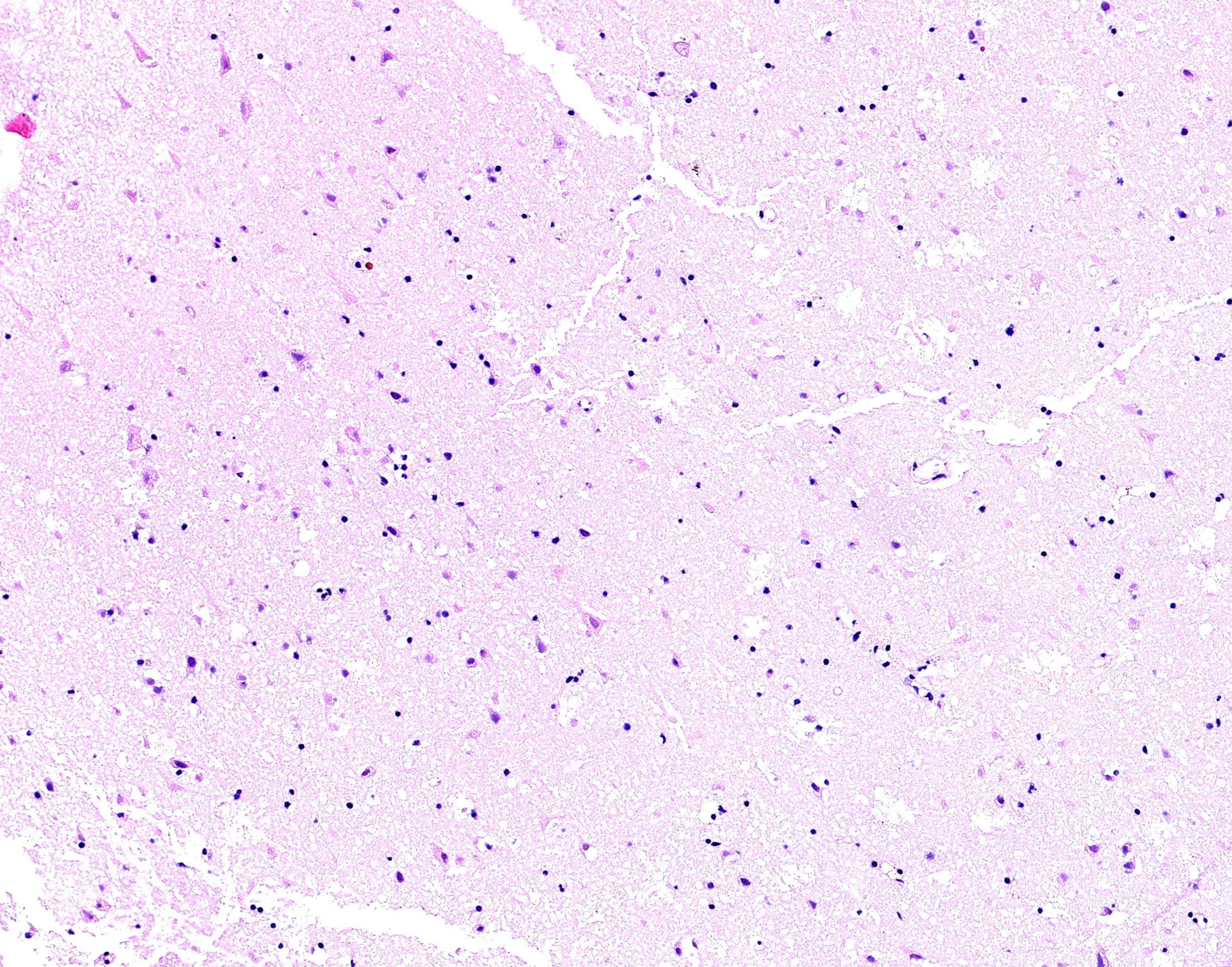

Supplement: Supplementary file 3 — Source Data Fig. 1 [file 44321_2024_39_MOESM3_ESM.zip › Figure 1/B/Parietal J12 MERGE_TileScan 3 Merged_Crop001_ch00_SV.jpg]

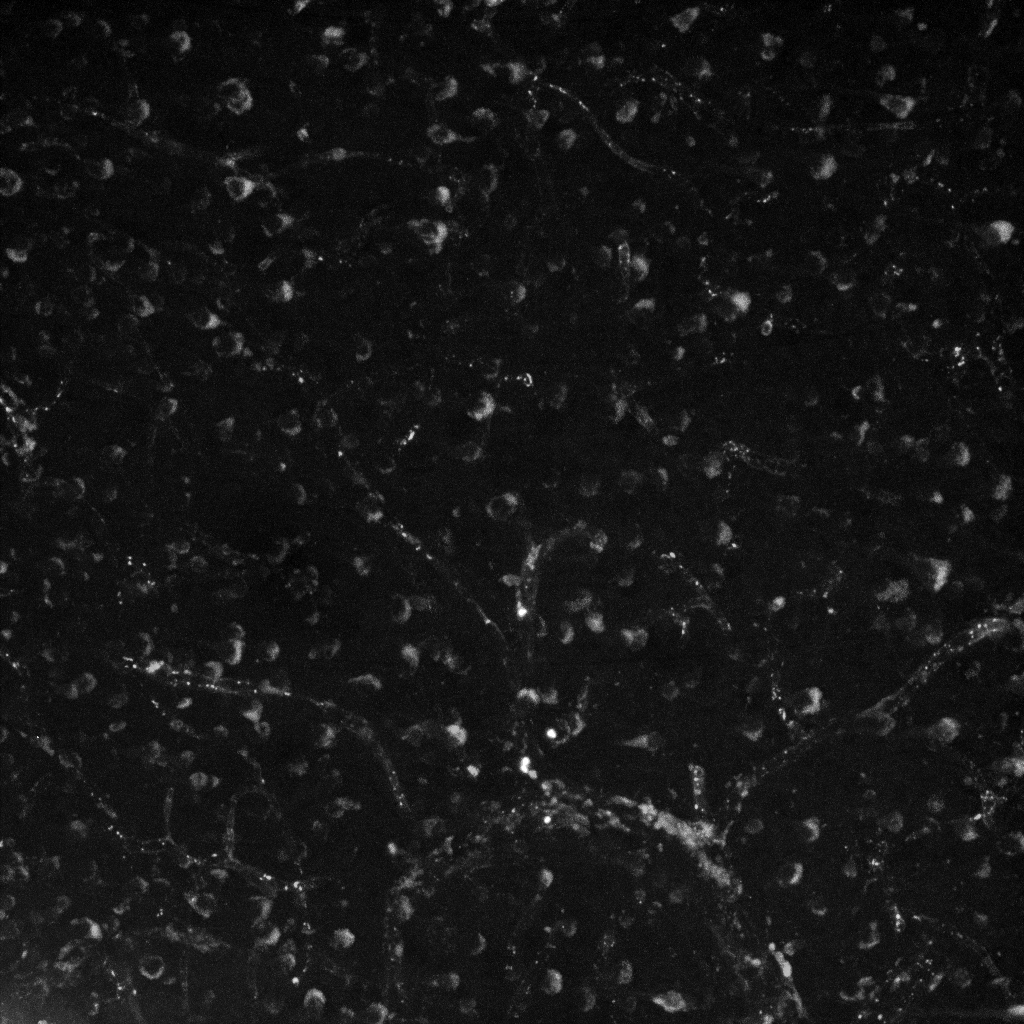

Supplement: Supplementary file 4 — Source Data Fig. 2 [file 44321_2024_39_MOESM4_ESM.zip › Figure 2/C/MAX_B1_1_F1.ims - B1_1_F1.ims Resolution Level 1 - C=1 b.tif]

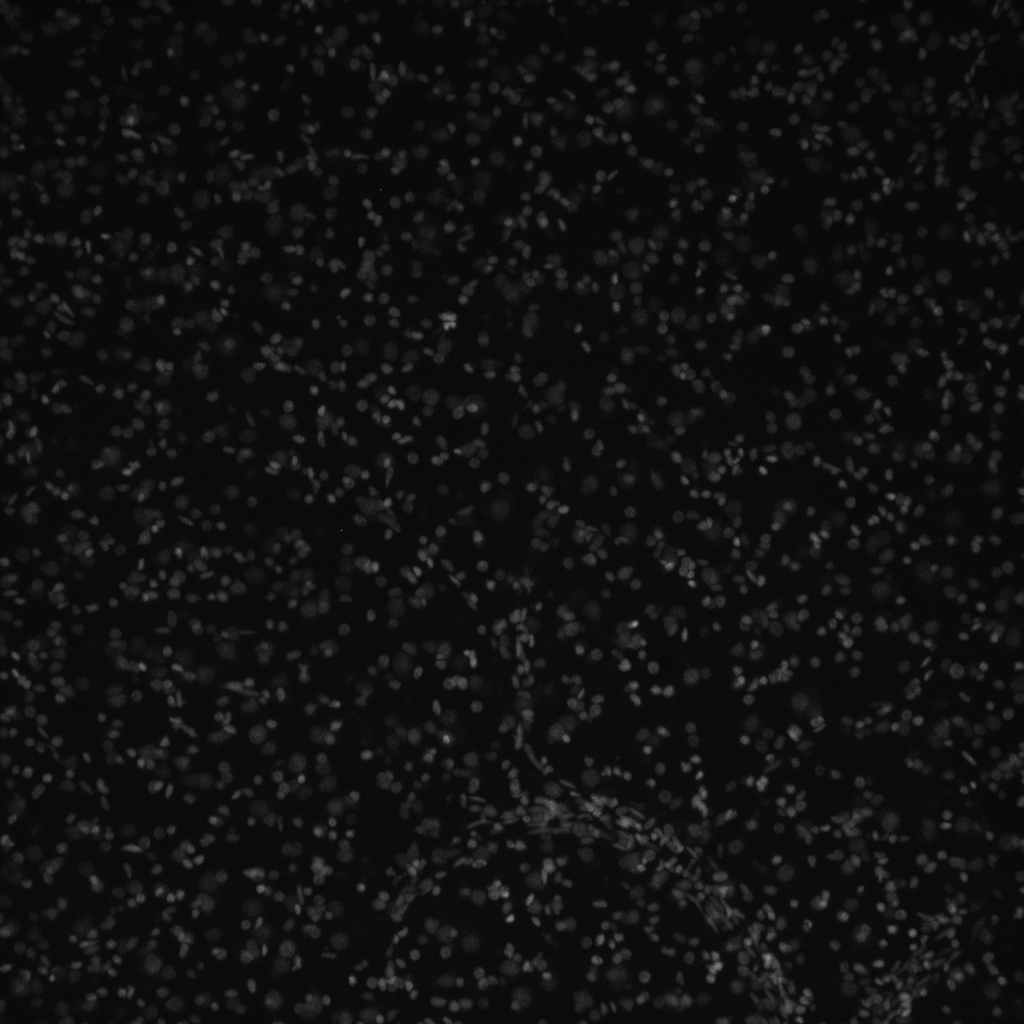

Supplement: Supplementary file 4 — Source Data Fig. 2 [file 44321_2024_39_MOESM4_ESM.zip › Figure 2/C/MAX_B1_1_F1.ims - B1_1_F1.ims Resolution Level 1 - C=0 b.tif]

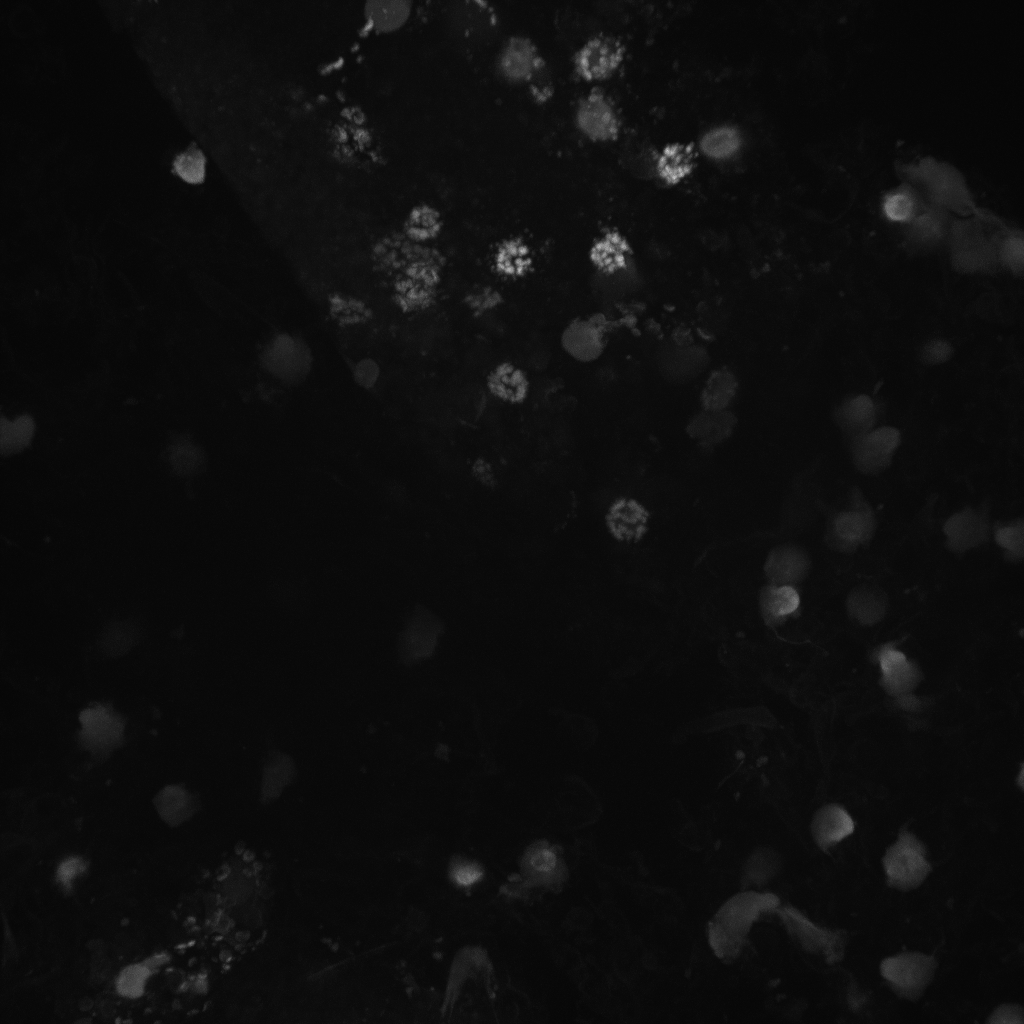

Supplement: Supplementary file 4 — Source Data Fig. 2 [file 44321_2024_39_MOESM4_ESM.zip › Figure 2/D/MAX_DAPI.tif]

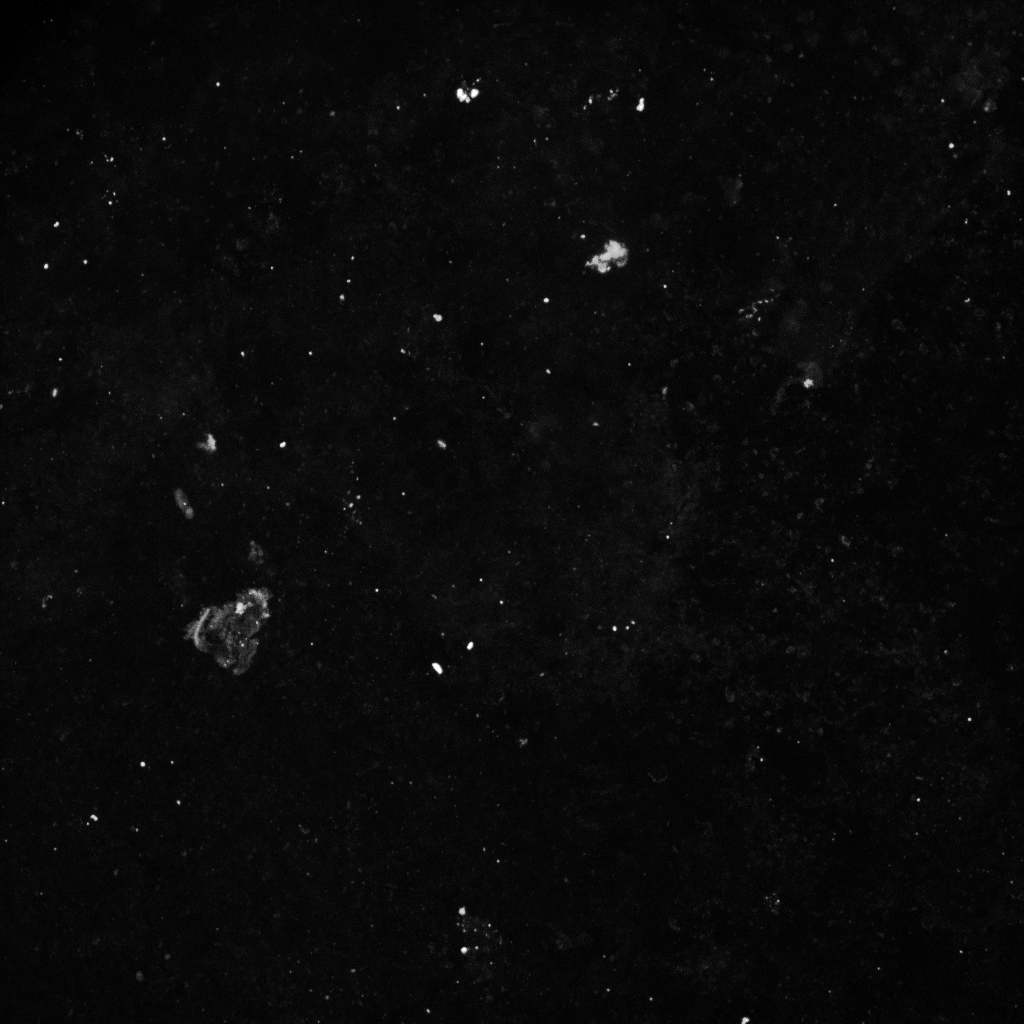

Supplement: Supplementary file 4 — Source Data Fig. 2 [file 44321_2024_39_MOESM4_ESM.zip › Figure 2/D/MAX_occ 20x_2020-10-12_2.ims - occ 20x_2020-10-12_2.ims Resolution Level 1 - C=1.tif]

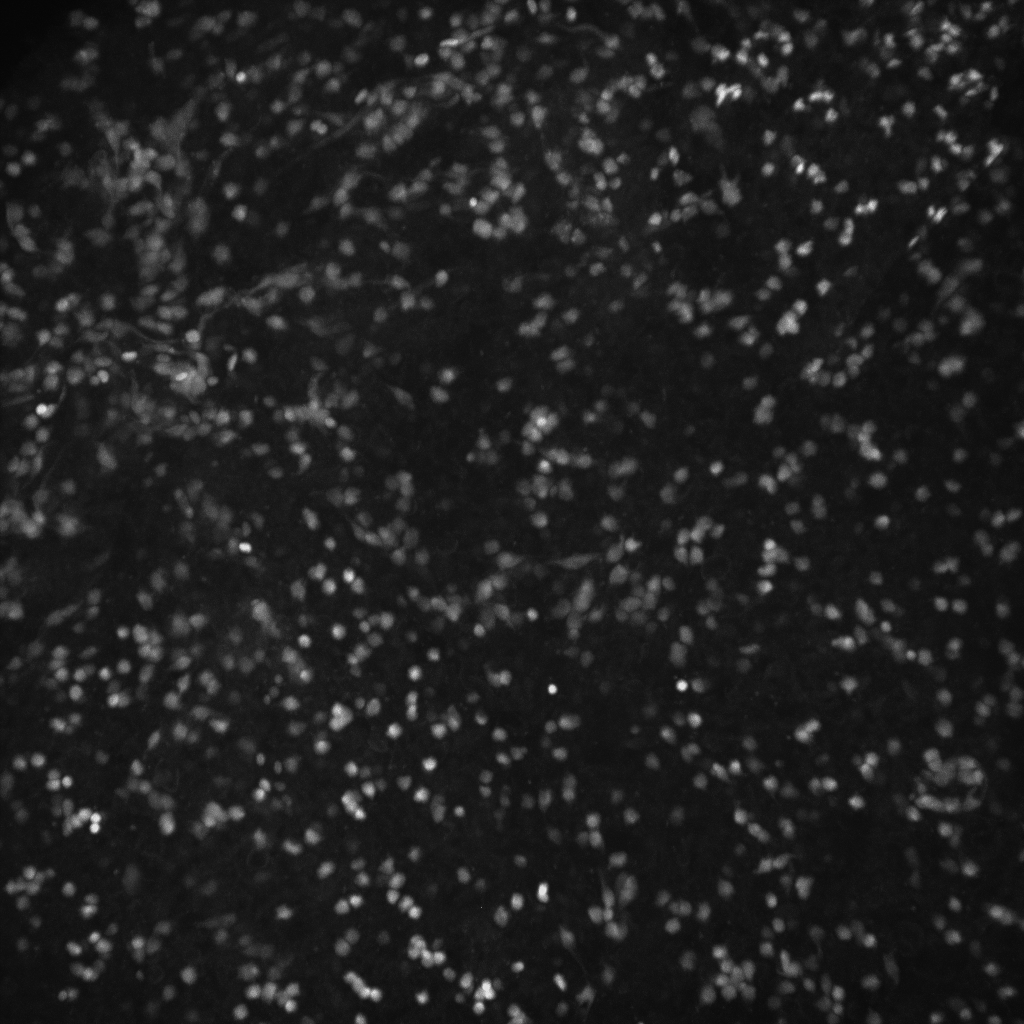

Supplement: Supplementary file 4 — Source Data Fig. 2 [file 44321_2024_39_MOESM4_ESM.zip › Figure 2/D/MAX_occ 20x_2020-10-12_2.ims - occ 20x_2020-10-12_2.ims Resolution Level 1 - C=0.tif]

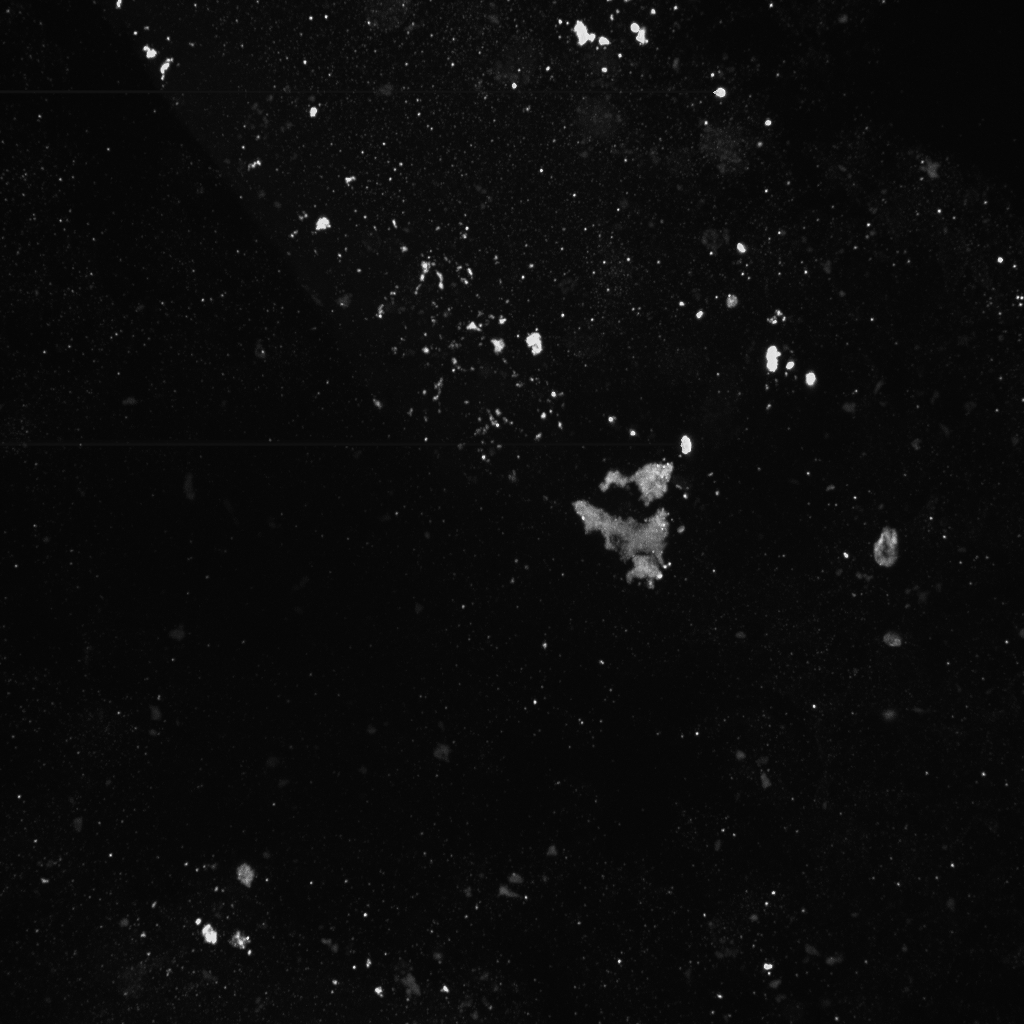

Supplement: Supplementary file 4 — Source Data Fig. 2 [file 44321_2024_39_MOESM4_ESM.zip › Figure 2/D/MAX_IBA-1.tif]

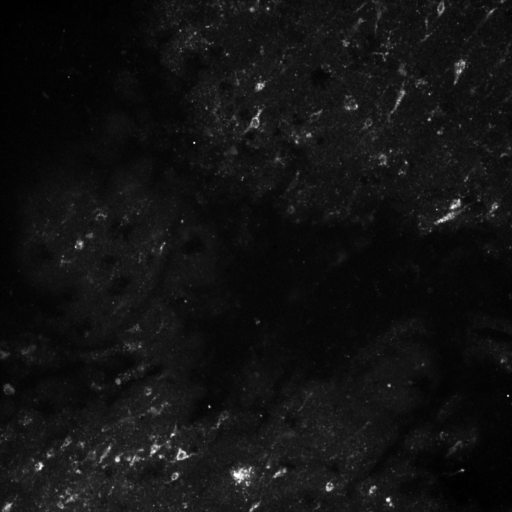

Supplement: Supplementary file 4 — Source Data Fig. 2 [file 44321_2024_39_MOESM4_ESM.zip › Figure 2/E/temp 20x_2020-10-15_1.ims - temp 20x_2020-10-15_1.ims Resolution Level 1 - C=0 b.tif]

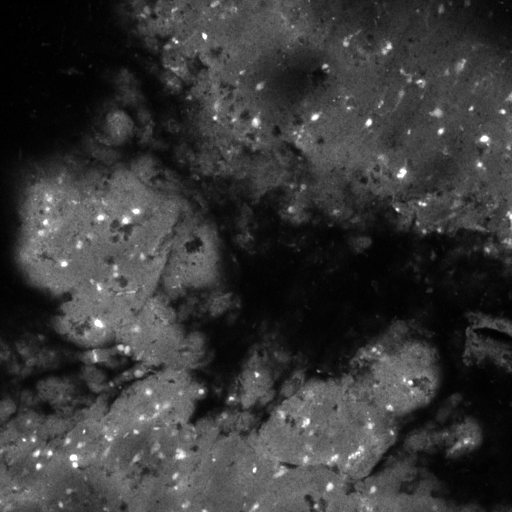

Supplement: Supplementary file 4 — Source Data Fig. 2 [file 44321_2024_39_MOESM4_ESM.zip › Figure 2/E/temp 20x_2020-10-15_1.ims - temp 20x_2020-10-15_1.ims Resolution Level 1 - C=1 b.tif]

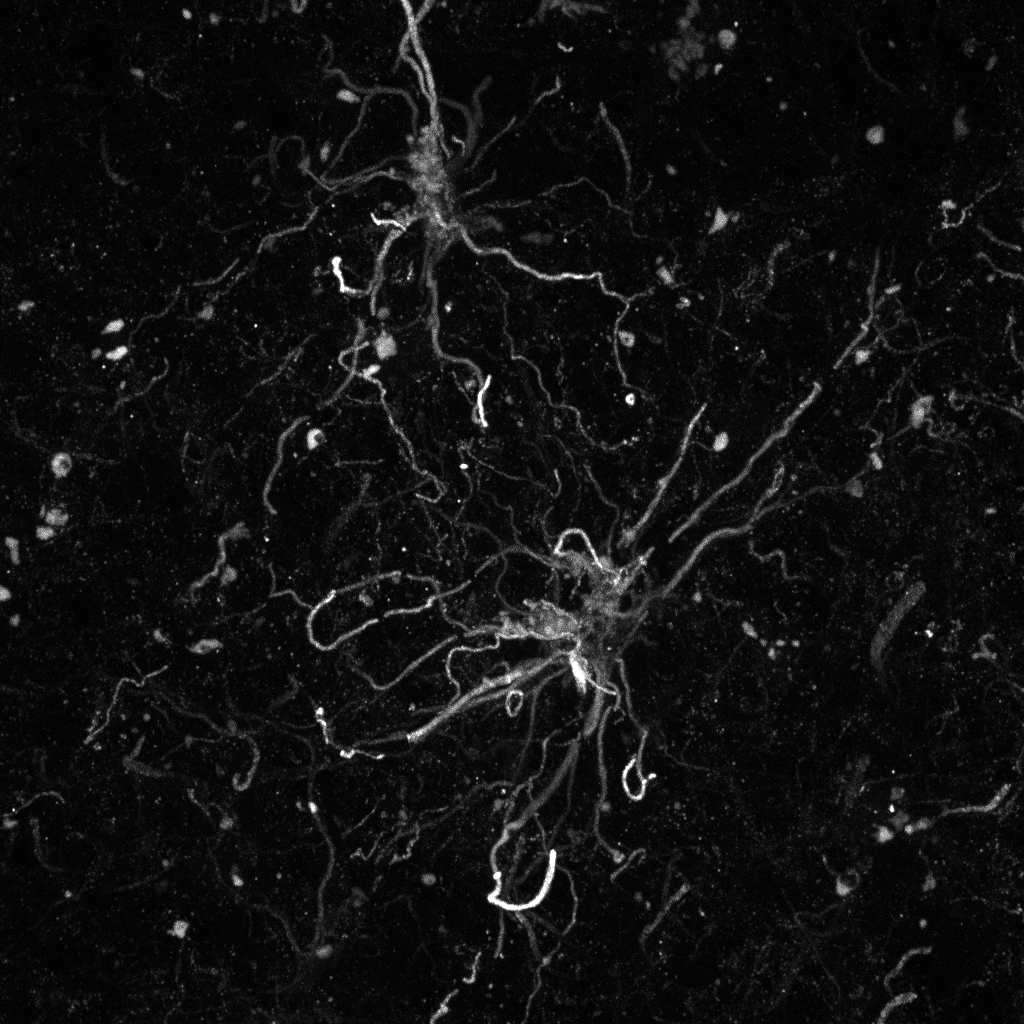

Supplement: Supplementary file 4 — Source Data Fig. 2 [file 44321_2024_39_MOESM4_ESM.zip › Figure 2/B/MAX_GFAP 100x b.tif]

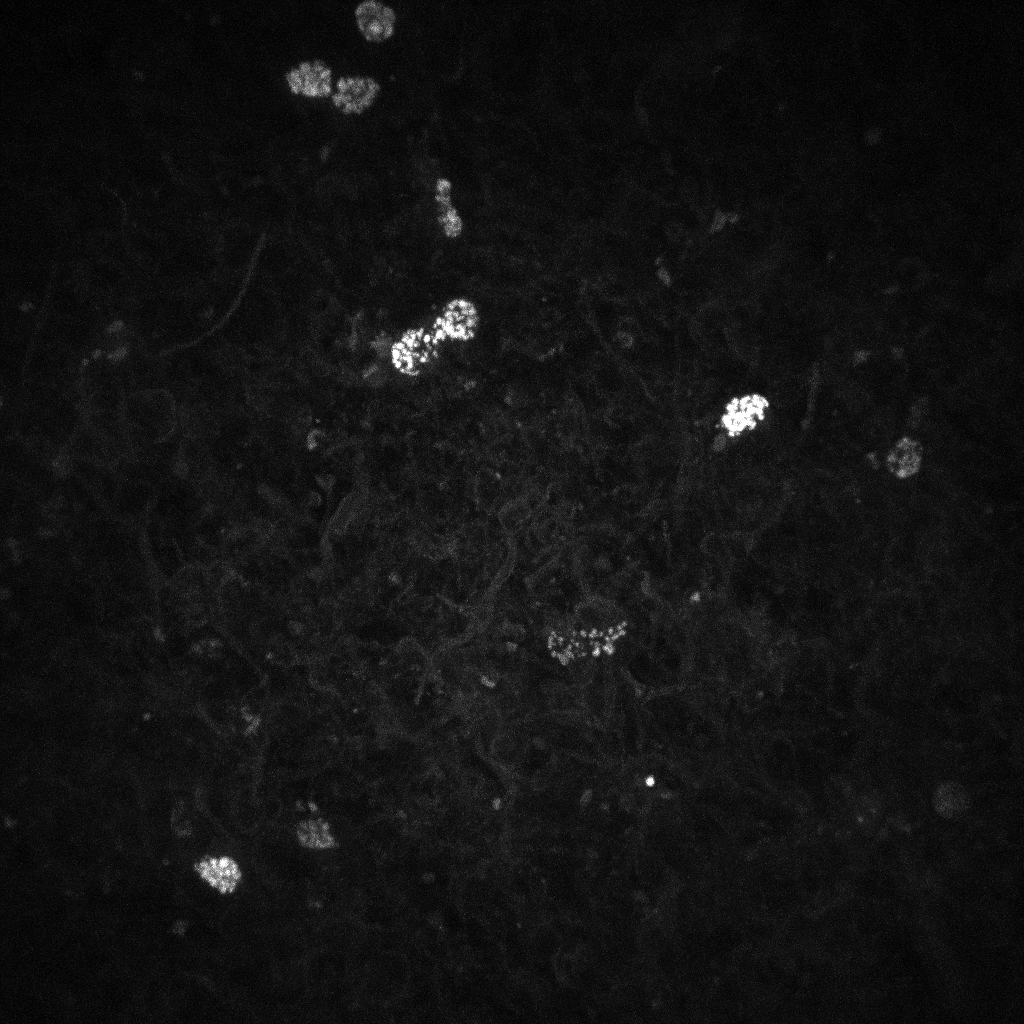

Supplement: Supplementary file 4 — Source Data Fig. 2 [file 44321_2024_39_MOESM4_ESM.zip › Figure 2/B/MAX_Dapi 100x b.tif]

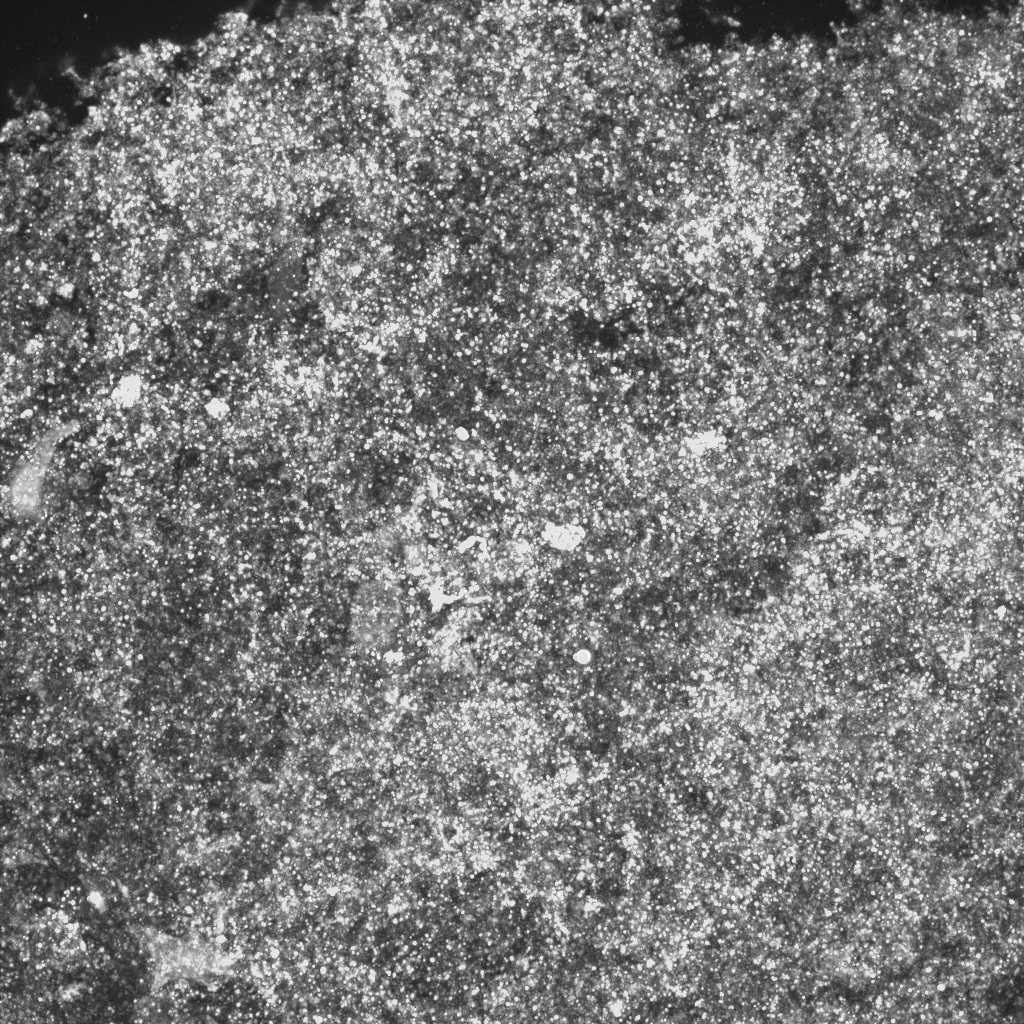

Supplement: Supplementary file 5 — Source Data Fig. 3 [file 44321_2024_39_MOESM5_ESM.zip › Figure 3/A-B/MAX_Bassoon.tif]

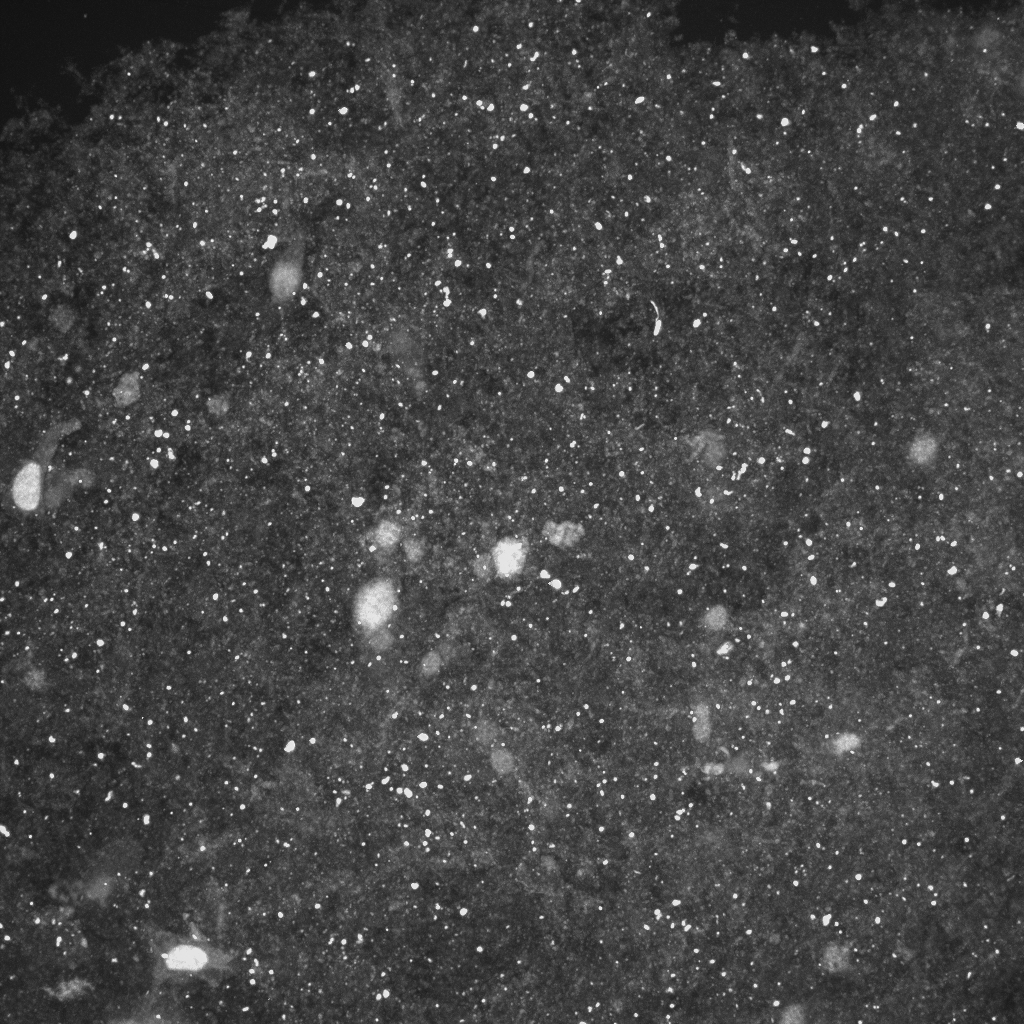

Supplement: Supplementary file 5 — Source Data Fig. 3 [file 44321_2024_39_MOESM5_ESM.zip › Figure 3/A-B/MAX_Homer-1.tif]

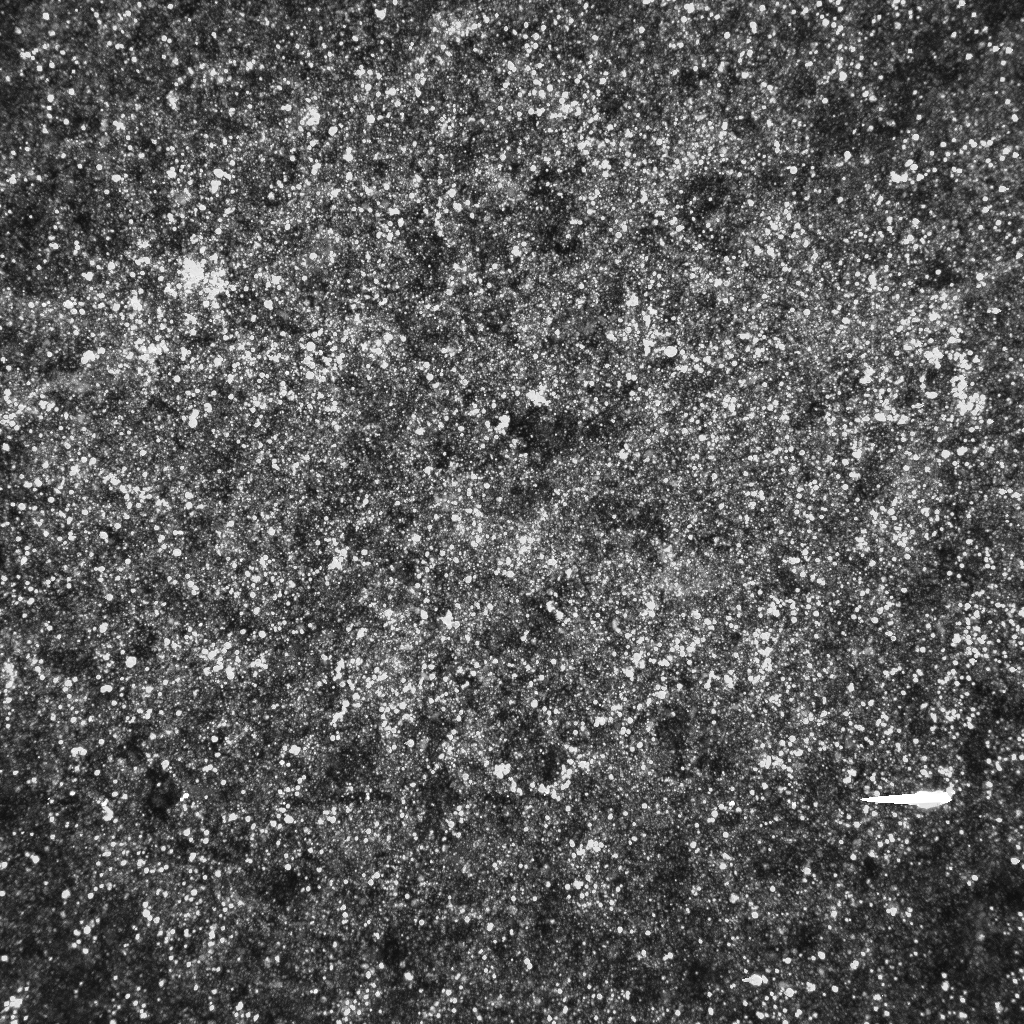

Supplement: Supplementary file 5 — Source Data Fig. 3 [file 44321_2024_39_MOESM5_ESM.zip › Figure 3/C/MAX_Bassoon.tif]

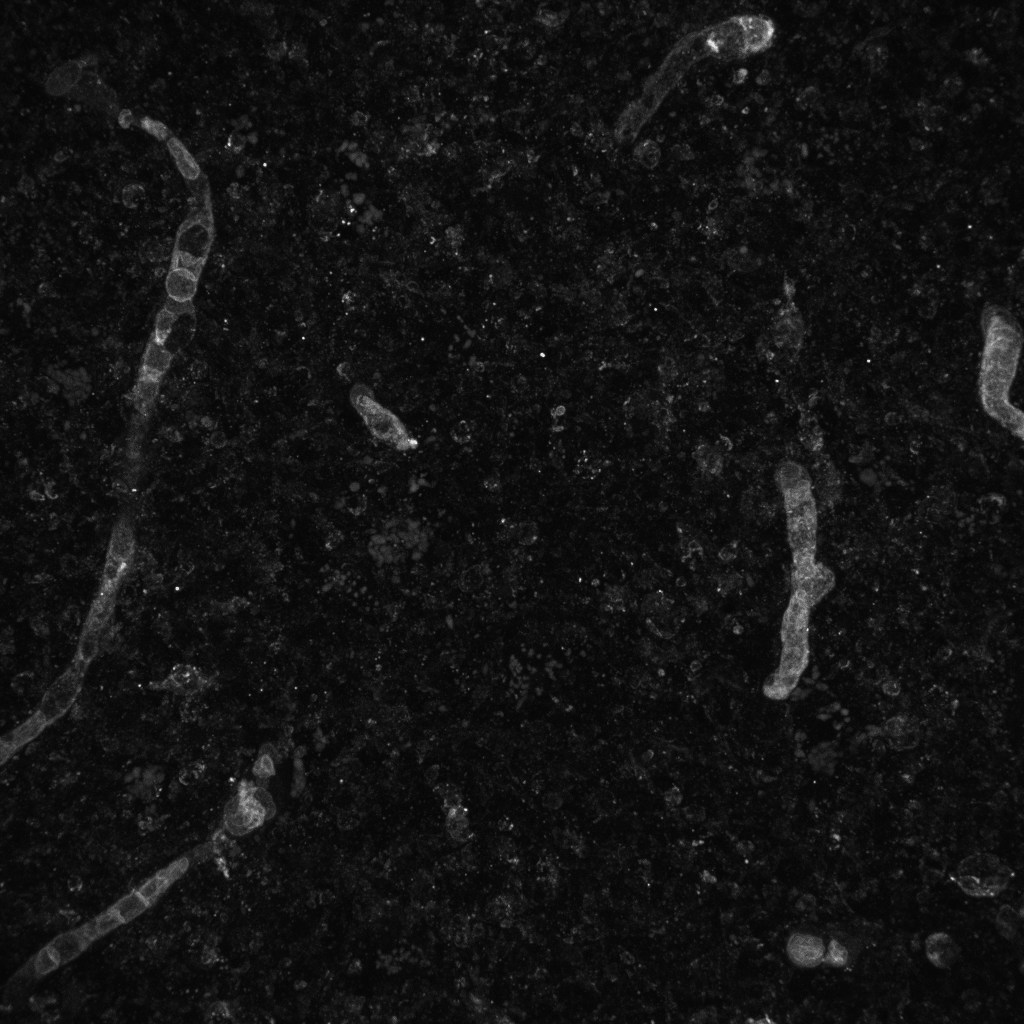

Supplement: Supplementary file 5 — Source Data Fig. 3 [file 44321_2024_39_MOESM5_ESM.zip › Figure 3/C/MAX_FLRT3.tif]

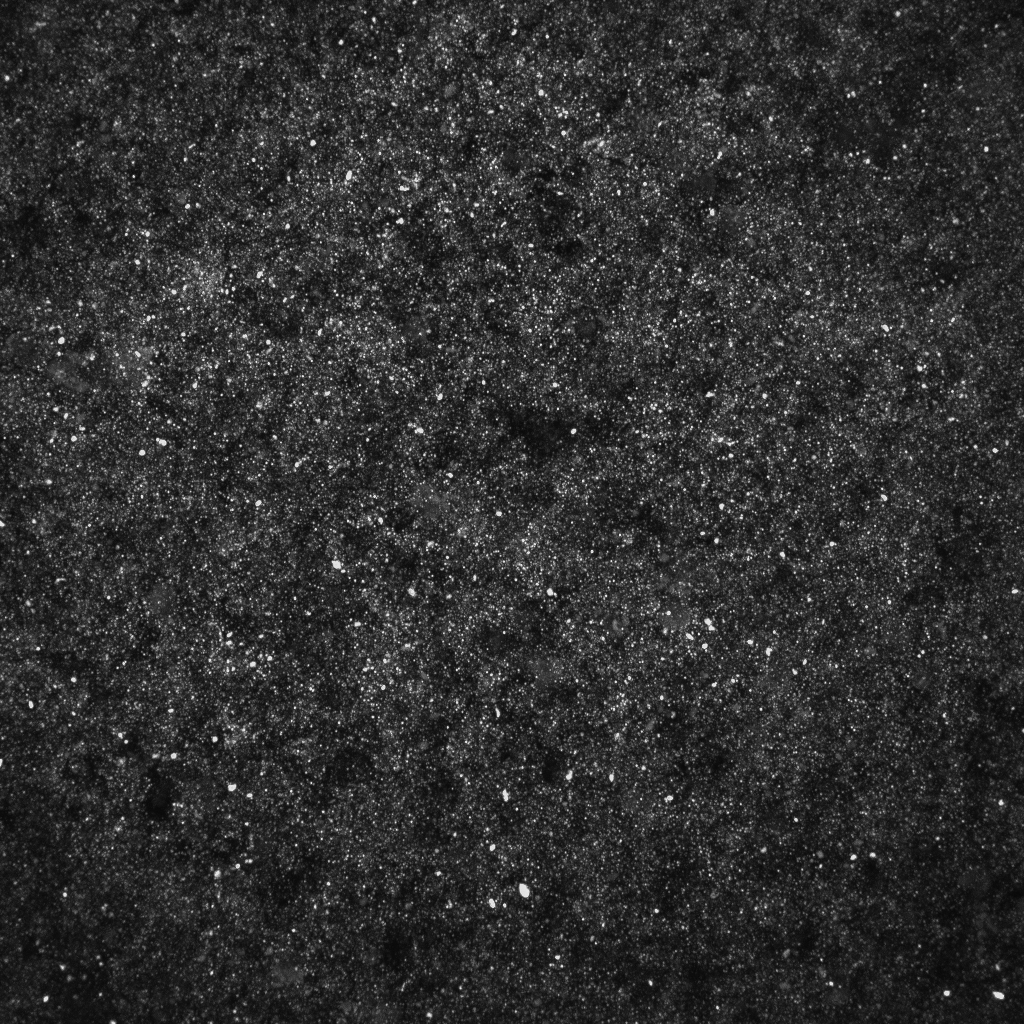

Supplement: Supplementary file 5 — Source Data Fig. 3 [file 44321_2024_39_MOESM5_ESM.zip › Figure 3/C/MAX_Homer-1.tif]

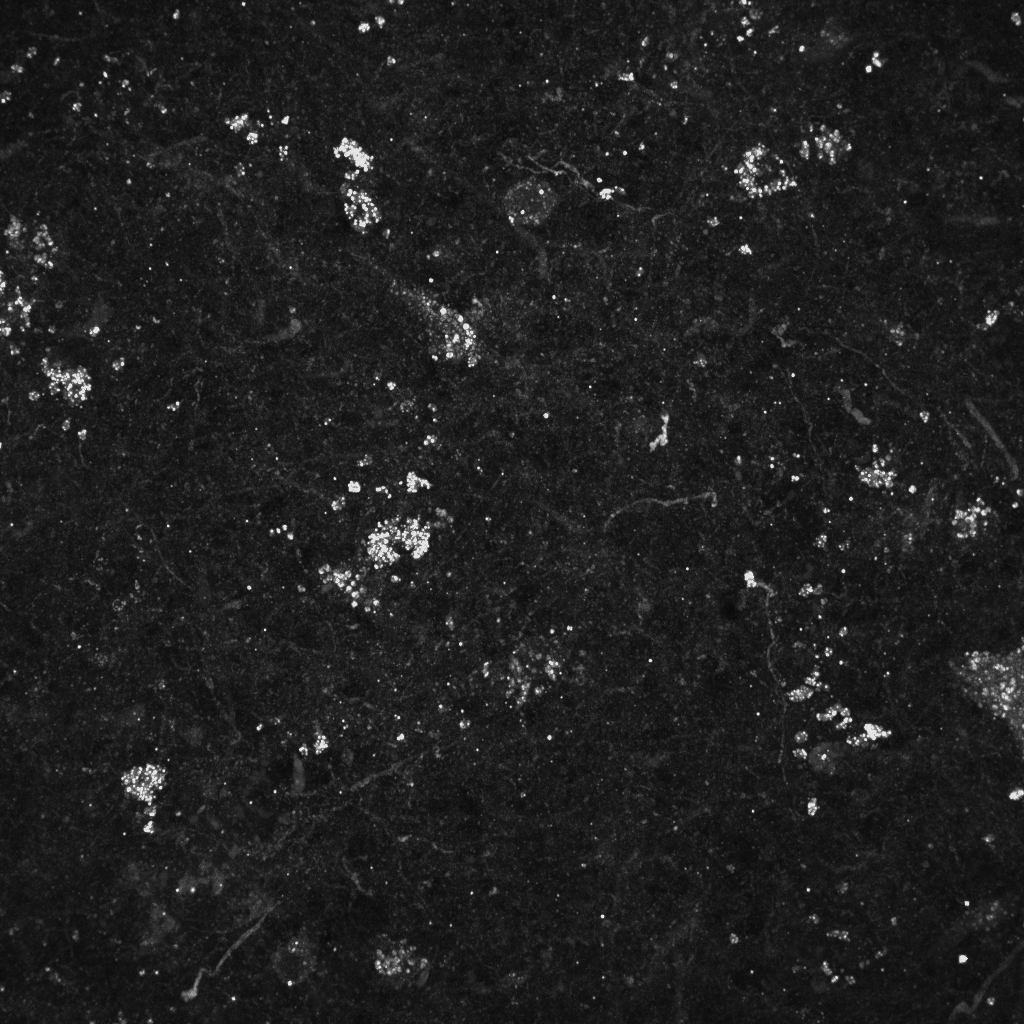

Supplement: Supplementary file 5 — Source Data Fig. 3 [file 44321_2024_39_MOESM5_ESM.zip › Figure 3/C/MAX_LPHN3.tif]

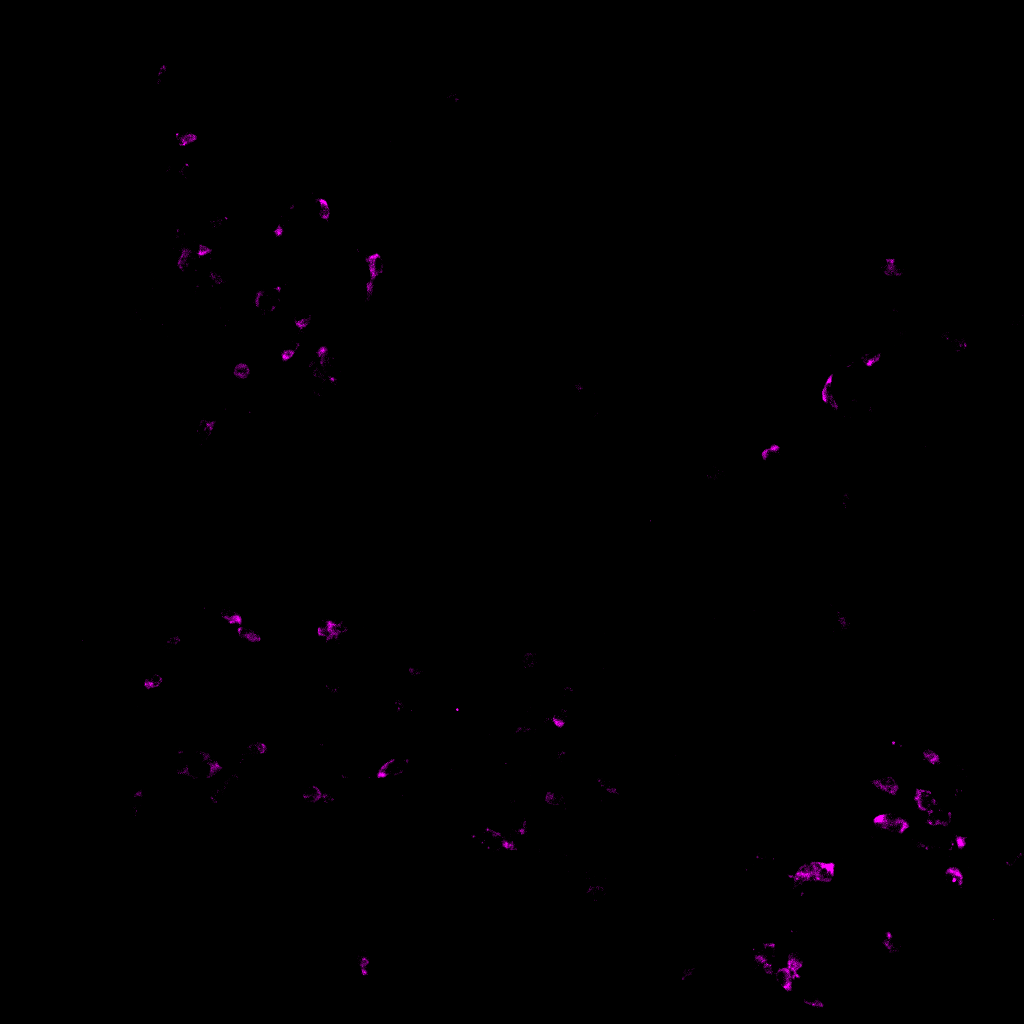

Supplement: Supplementary file 6 — Source Data Fig. 4 [file 44321_2024_39_MOESM6_ESM.zip › Figure 4/E/MOI 0.1 TAHV RNA.tif]

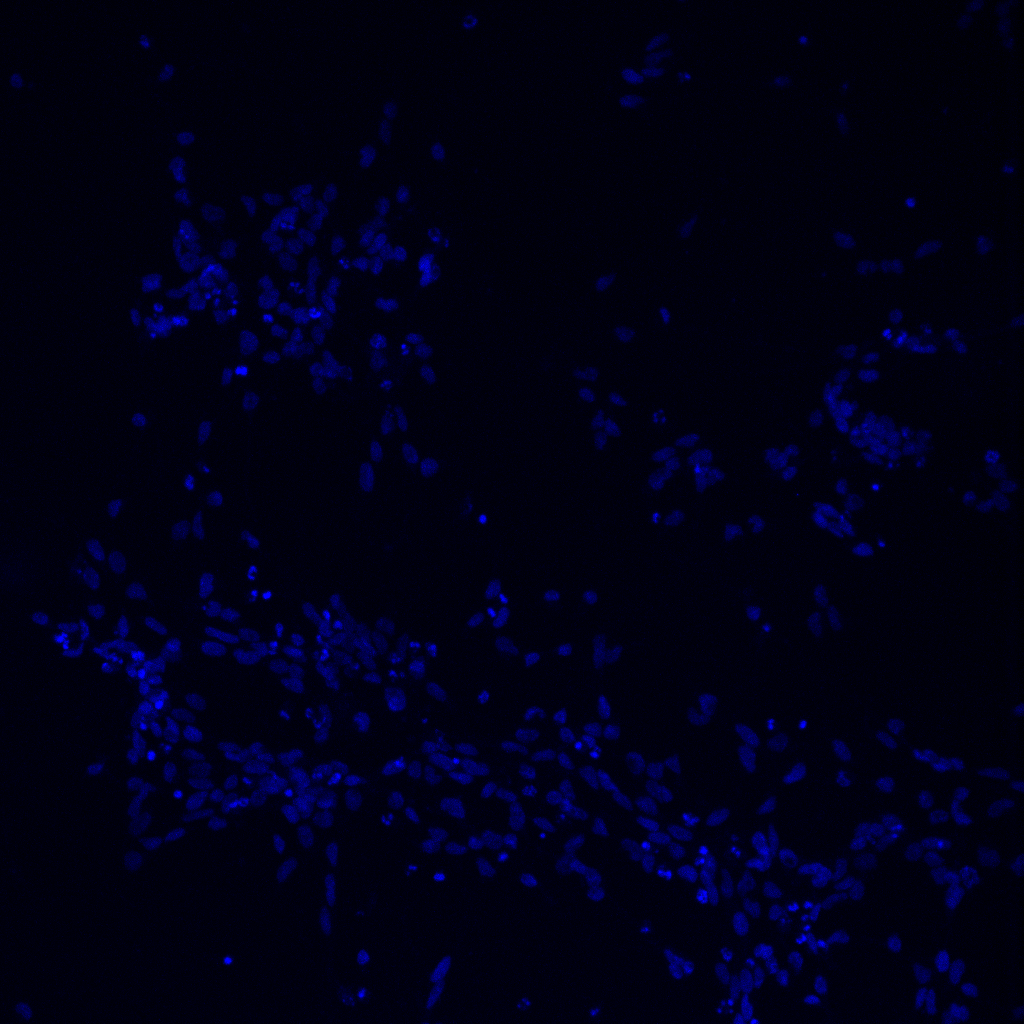

Supplement: Supplementary file 6 — Source Data Fig. 4 [file 44321_2024_39_MOESM6_ESM.zip › Figure 4/E/MOI 0.1 Dapi.tif]

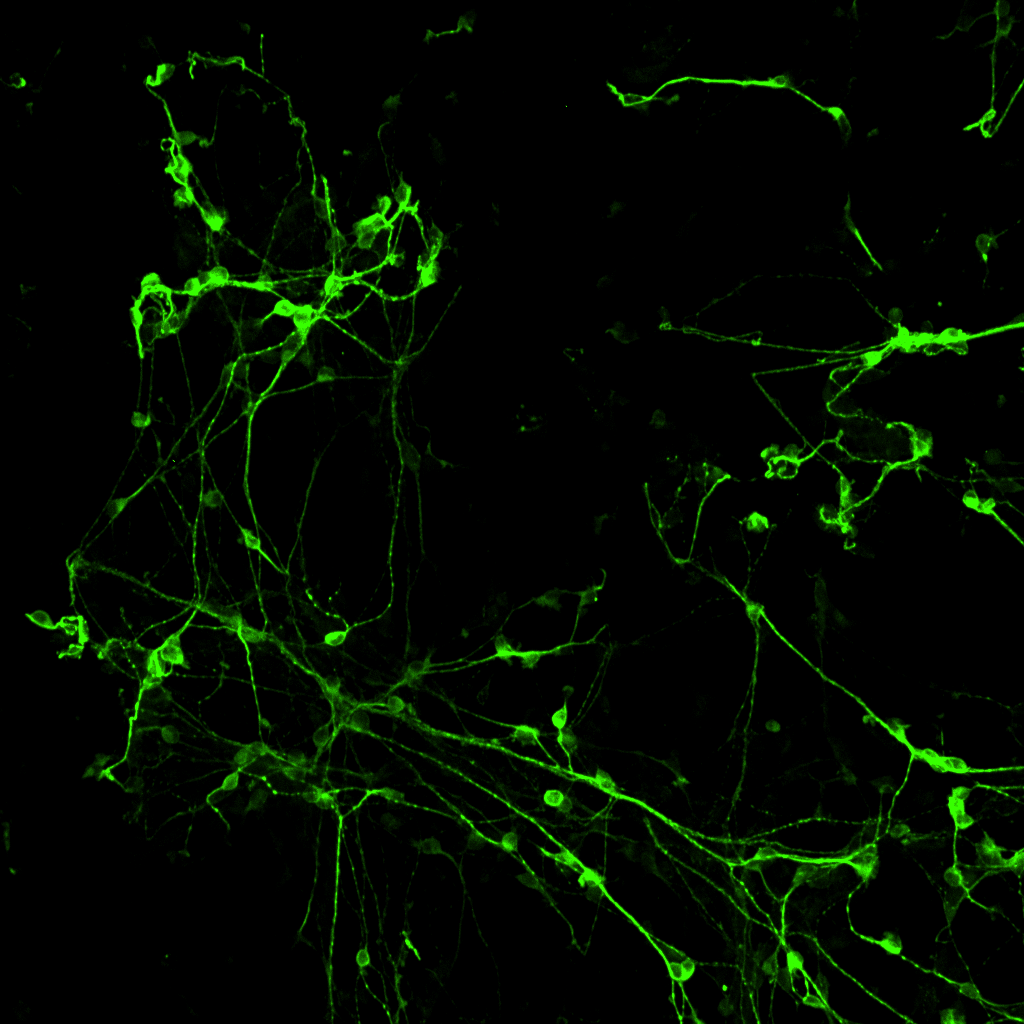

Supplement: Supplementary file 6 — Source Data Fig. 4 [file 44321_2024_39_MOESM6_ESM.zip › Figure 4/E/MOI 0.1 MAP2.tif]

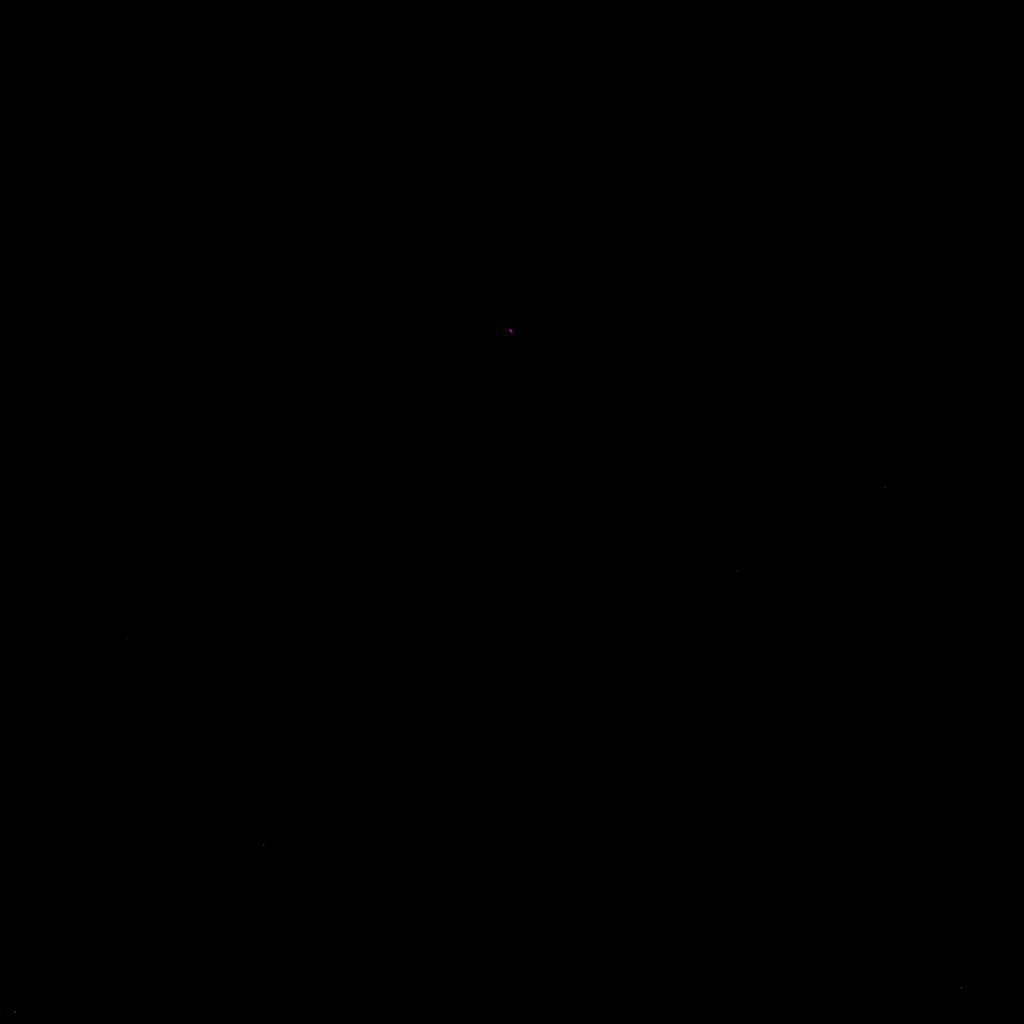

Supplement: Supplementary file 6 — Source Data Fig. 4 [file 44321_2024_39_MOESM6_ESM.zip › Figure 4/E/NI TAHV RNA.tif]

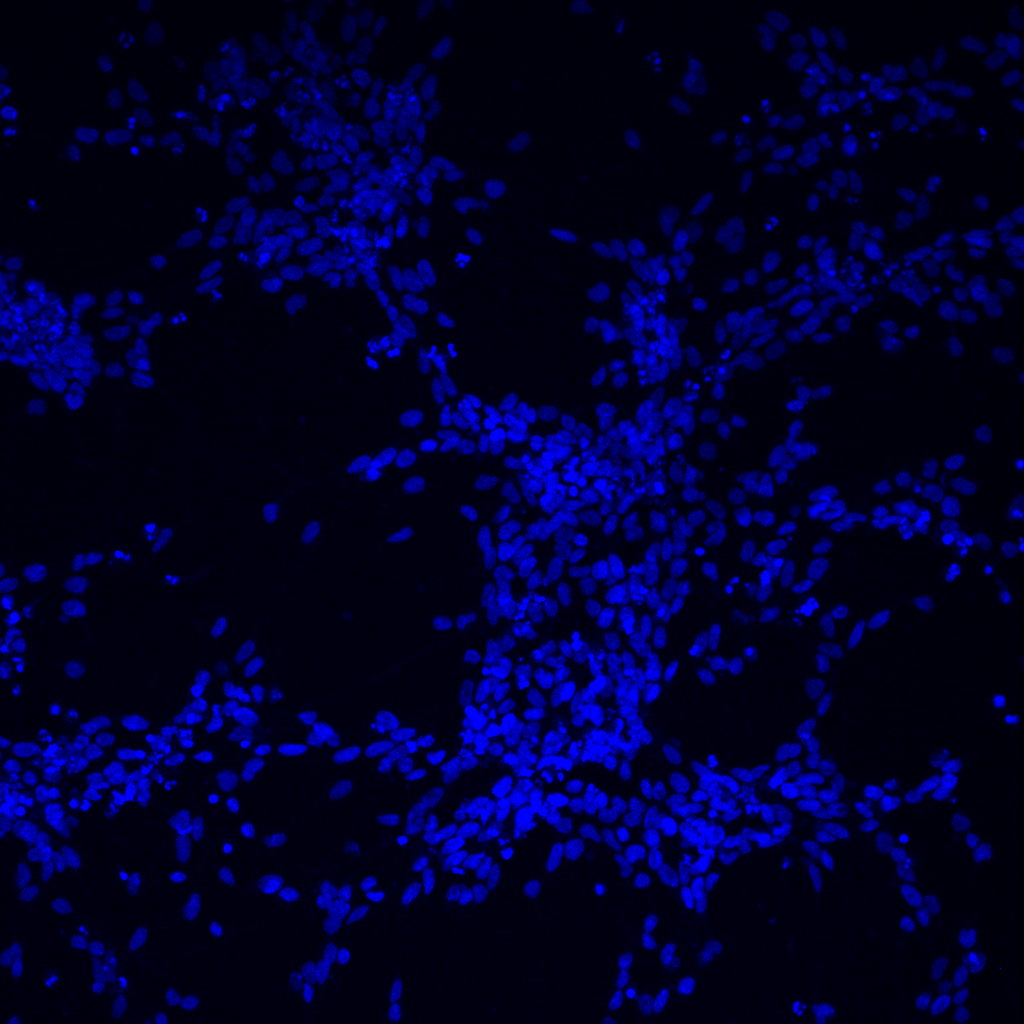

Supplement: Supplementary file 6 — Source Data Fig. 4 [file 44321_2024_39_MOESM6_ESM.zip › Figure 4/E/NI Dapi.tif]

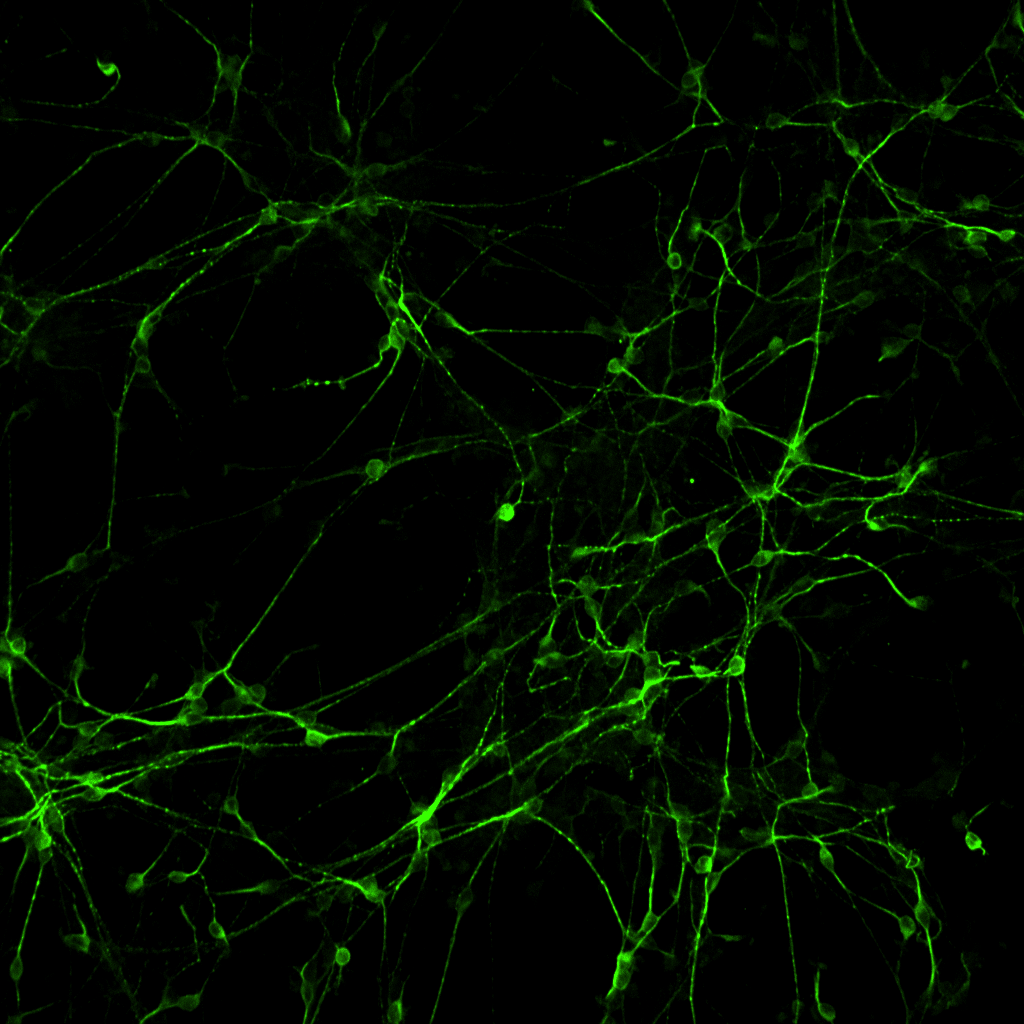

Supplement: Supplementary file 6 — Source Data Fig. 4 [file 44321_2024_39_MOESM6_ESM.zip › Figure 4/E/NI MAP2.tif]

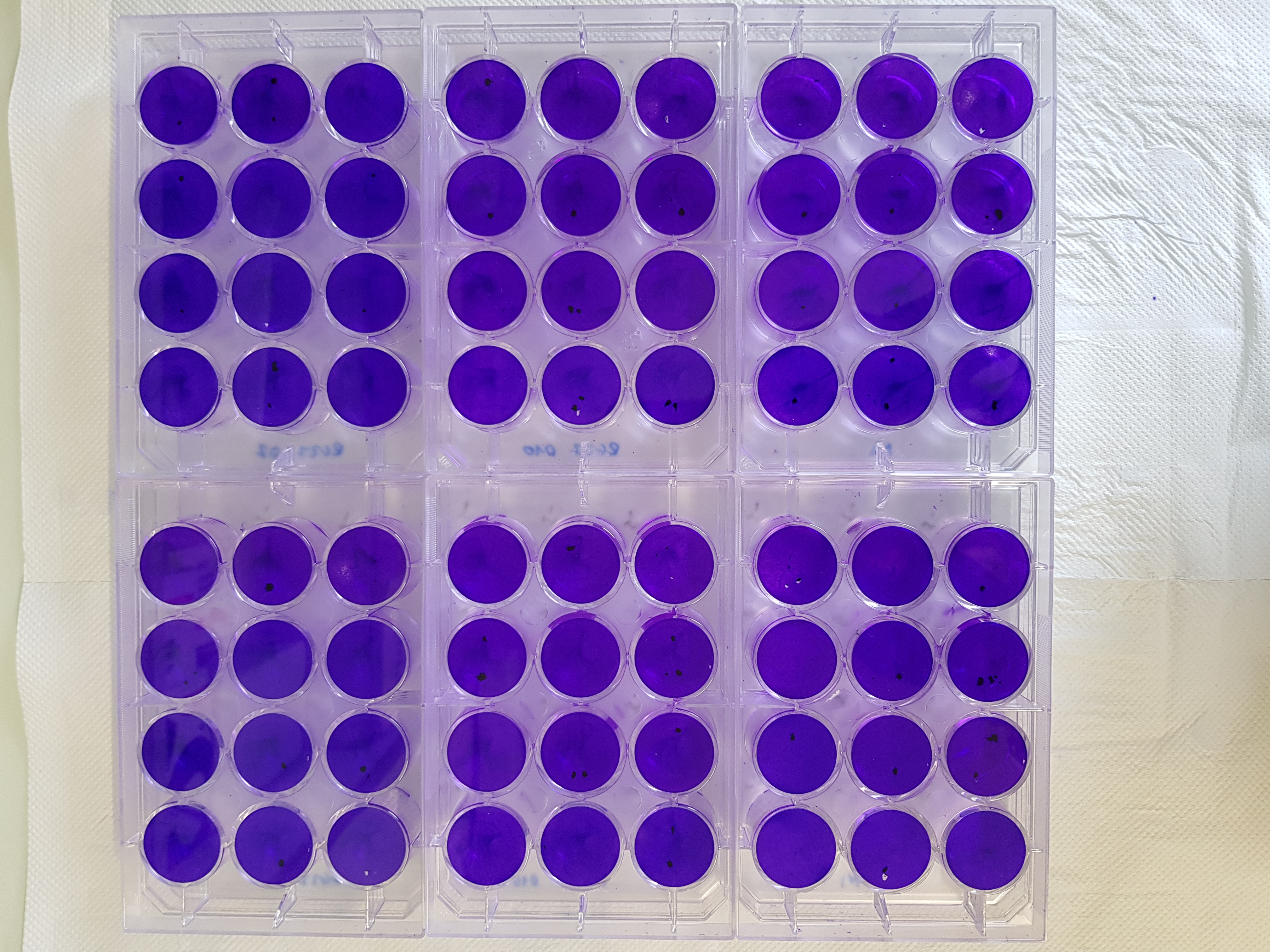

Supplement: Supplementary file 7 — Source Data Fig. 5 [file 44321_2024_39_MOESM7_ESM.zip › Figure 5/D/5D Brain slice plaque assay.jpg]

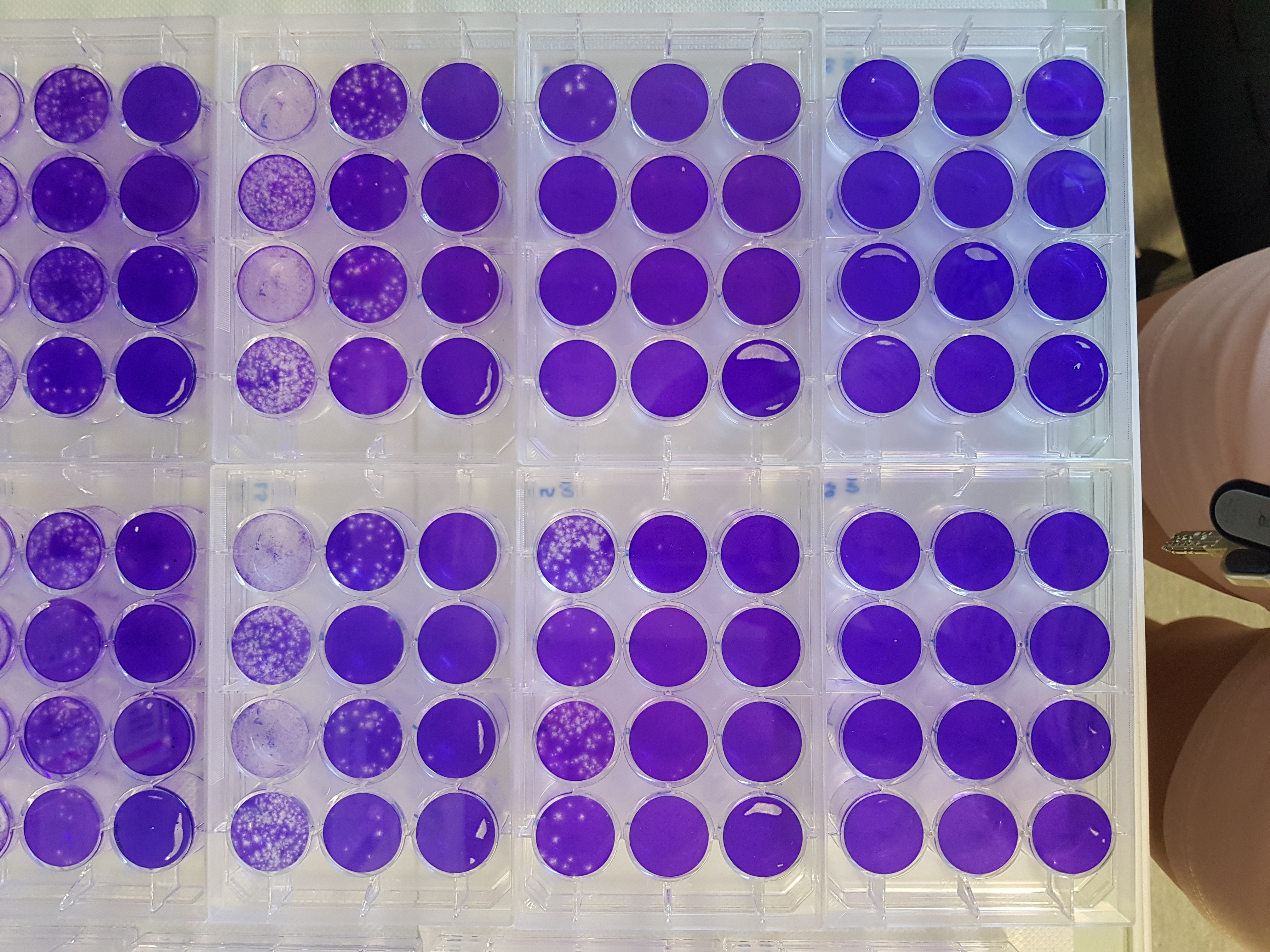

Supplement: Supplementary file 7 — Source Data Fig. 5 [file 44321_2024_39_MOESM7_ESM.zip › Figure 5/D/5D VeroE6 plaque assay.jpg]
