# Supplementary material for: Organotypic culture of human brain explants as a preclinical model for AI-driven antiviral studies
Source: EMBO Mol Med. 2024 Mar 12;16(4):1004–26. doi: 10.1038/s44321-024-00039-9 (PMC11018746; doi:10.1038/s44321-024-00039-9)
Supplement: Supplementary file 10 — Expanded View Figures [file 44321_2024_39_MOESM10_ESM.pdf]

Expanded View Figures

Figure EV1. Immunohistochemistry of OPAB.

(**A**, **B**) Immunochemistry of Hemalin-Eosin (H&E), neurons (NeuN), oligodendrocytes (Oligo2) and astrocytes (GFAP) from parietal (**A**) or frontal (**B**) areas of OPAB at indicated days in vitro (DIV). The condition 0 DIV correspond to immediate sample collection at autopsy, before vibratome slicing and OPAB. The black squares correspond to the zoomed area on the right panels. Images were acquired on Leica Thunder using 20x magnification. Scale bar: 100 µm.

© The Author(s)

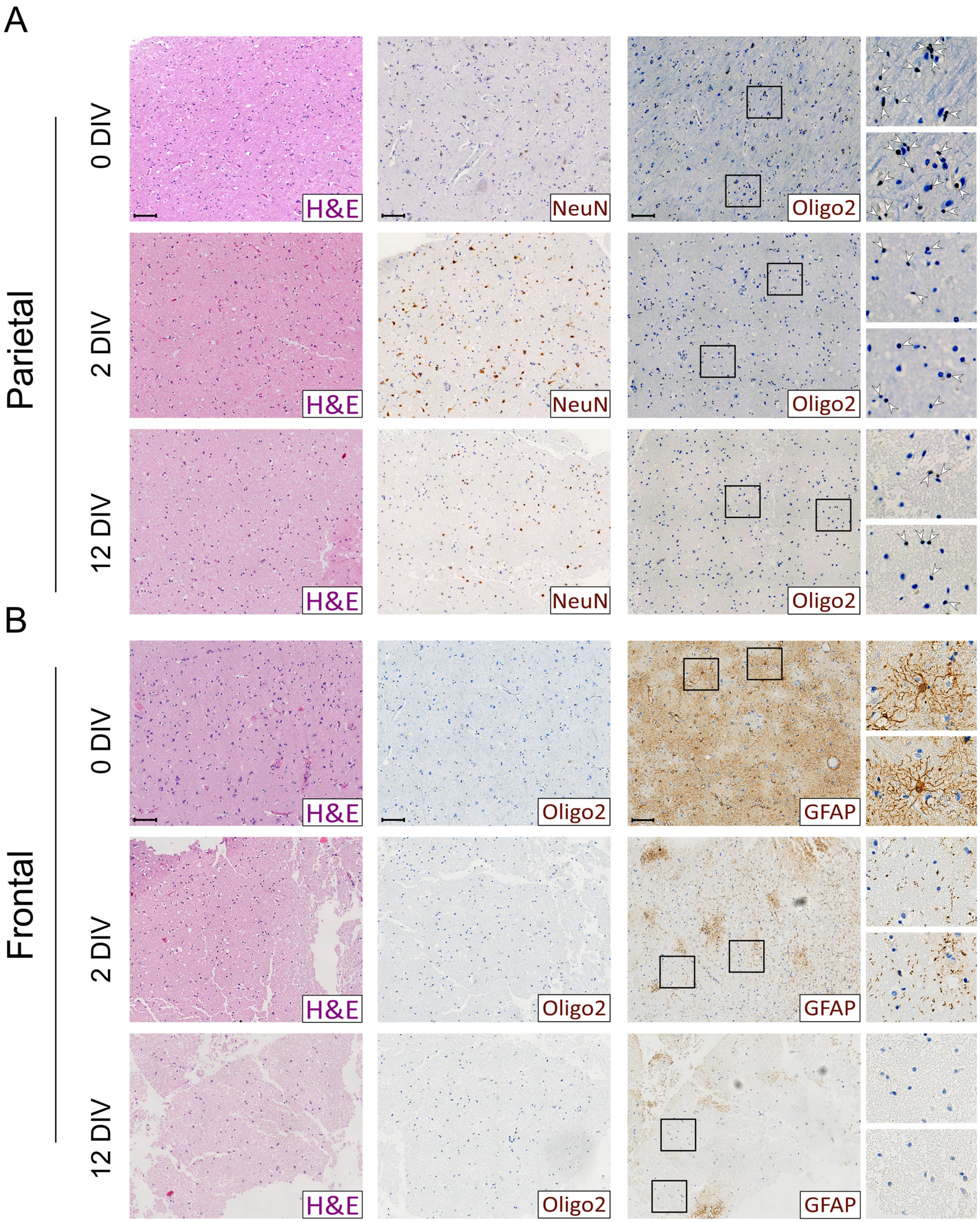
A
Parietal
0 DIV
2 DIV
12 DIV
H&E
NeuN
Oligo2
B
Frontal
0 DIV
2 DIV
12 DIV
H&E
Oligo2
GFAP

 © The Author(s)

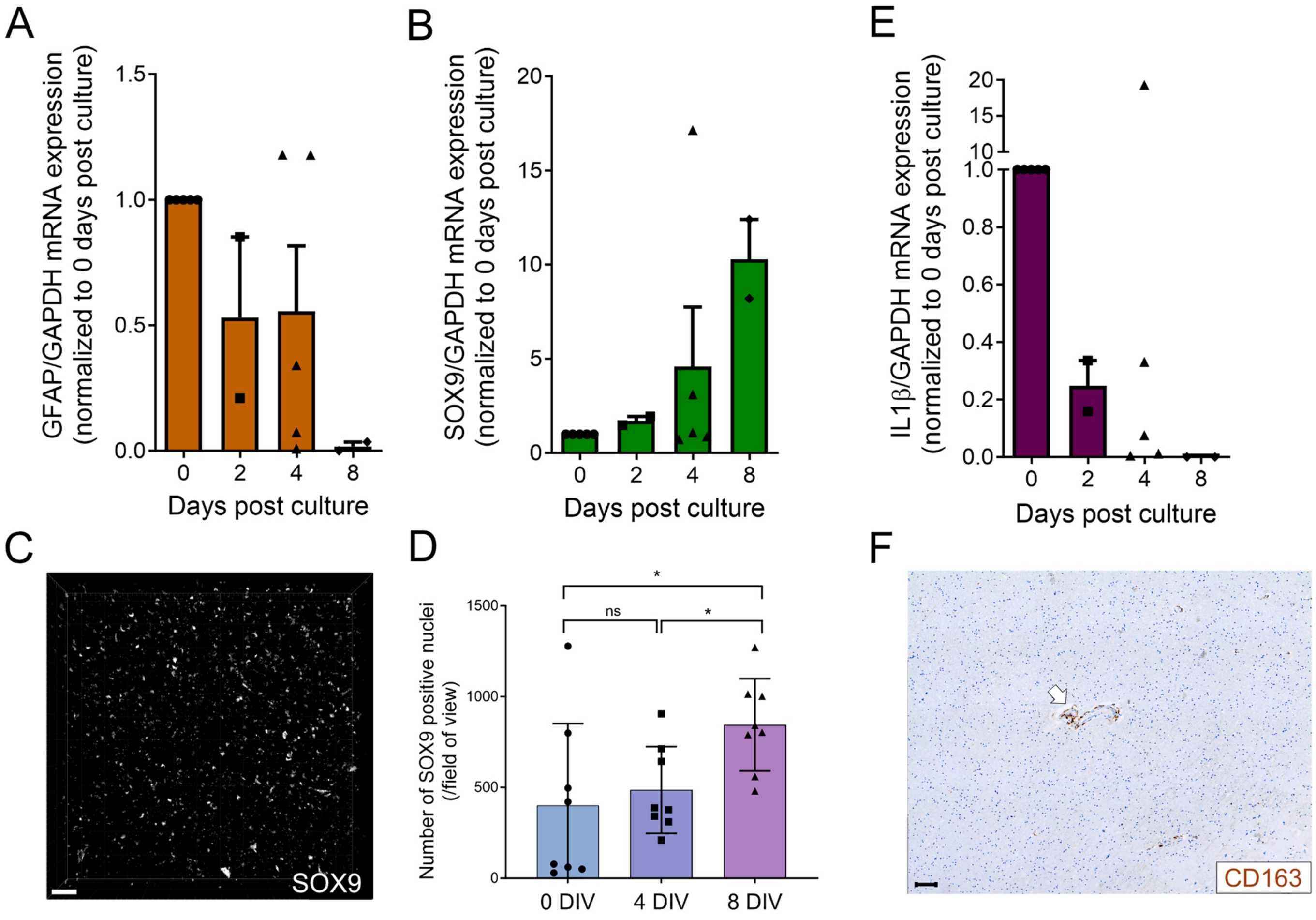

Figure EV2. Characterization of astrocytic and immunoreactivity of OPAB.

(**A**, **B**) OPAB were cultured for indicated days in vitro, RNA was extracted and RT-qPCR was performed to measure the expression levels of GFAP- (**A**) and SOX9-coding (**B**) mRNA over time. Data are mean +/− SEM and each dot corresponds to the measurement of the mRNA levels per slice, coming from at least two donors. (**C, D**) OPAB were fixed at 4 DIV (**C**) or indicated times. (**D**) and stained with anti-SOX9 antibody, labeling the nucleus of astrocytes. (**C**) Representative 3D confocal image of SOX9 staining. Scale bar: 50 μm. (**D**) Quantification of the number of SOX9-positive nuclei per field of view at indicated time post in vitro culture. Unpaired t-test p value < 0.05 (*); ns: non-significant. Data are mean +/− SD from 8 fields of view taken from two slices from two donors. (**E**) Samples processed as in A-B were assessed for expression of IL1β-coding mRNA. Of note, one slice at day 4 post culture exhibit oddly high values preventing reliable statistical analysis. Data are mean +/− SEM and each dot corresponds to the measurement of the mRNA levels per slice, coming from at least two donors. (**F**) Immunohistochemistry of OPAB at 0 DIV showing absence of monocyte/macrophage/microglia (CD163, brown) staining in the cortex. The white arrow highlights a blood vessel at which CD163 staining is elevated, indicating that monocyte/macrophages did not extensively infiltrated the cortex.

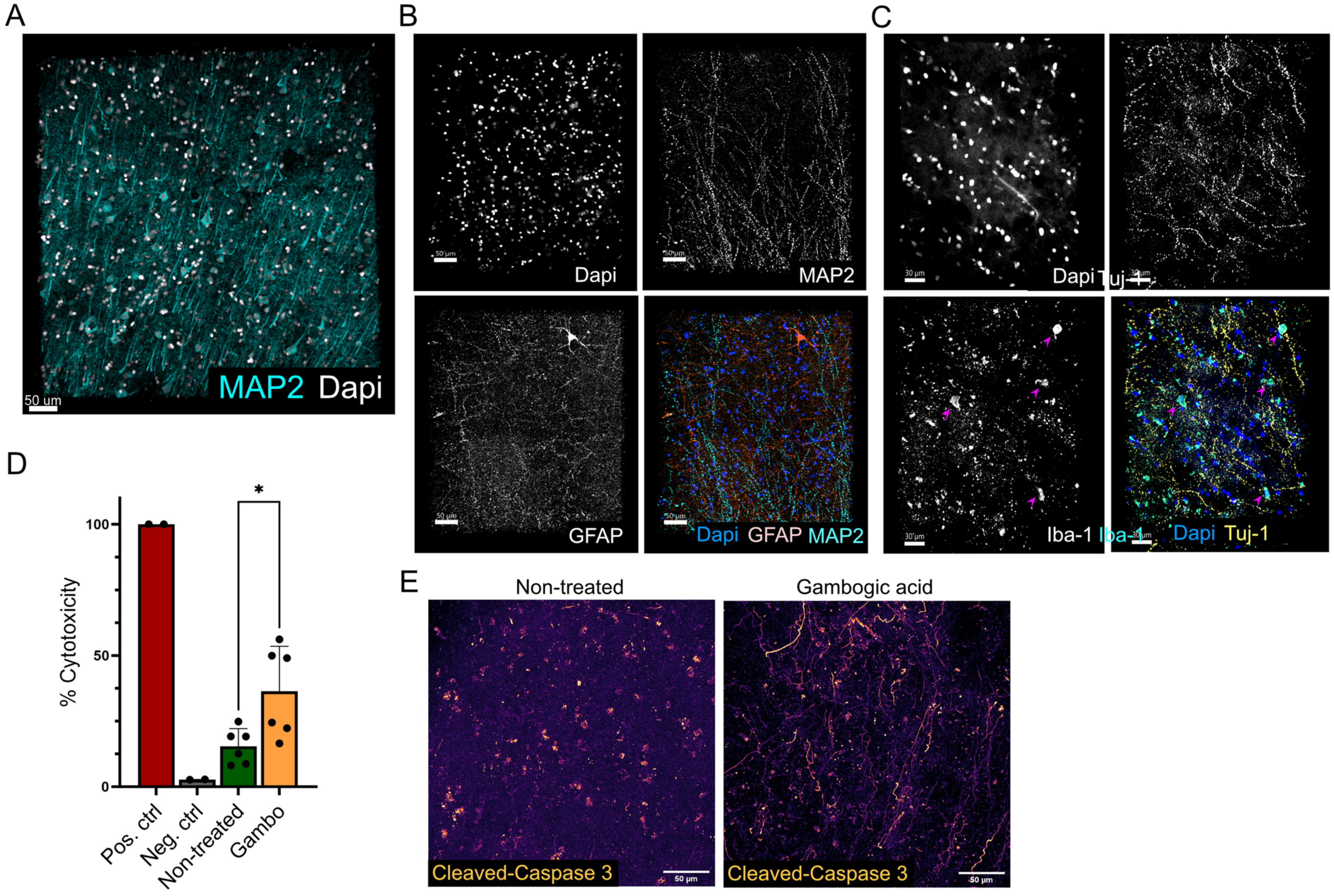

Figure EV3. Characterization of OPAB by immunofluorescence.

(**A**) Snapshots from 3D confocal imaging (top view) of frontal OPAB, showing parallelly aligned post-mitotic neurons (MAP2 in cyan) and nuclei (Dapi in white). Scale bar: 50 µm. (**B**, **C**) Three-dimensional imaging of neurons (Tuj1 and MAP2), astrocytes (GFAP) and microglial cells (Iba1) from a frontal OPAB at 5 DIV. (**D**) Neuronal LUHMES cells treated for 24 h with 10 µg/ml Gambogic acid (Gambo), and cytotoxicity was measured in the supernatant using LDH assay. Unpaired t-test *p* value < 0.05 (*). Data are mean +/− SD from 2 individual experiments performed in triplicates. Controls were performed once per experiment. (**E**) OPAB were treated for 24 h with 10 µg/ml Gambogic acid and anti-Cleaved-Caspase-3 antibody labeling was performed. Confocal snapshots highlight brighter Cleaved-Caspase-3 staining upon Gambogic acid treatment. Scale bar: 50 µm.

 © The Author(s)

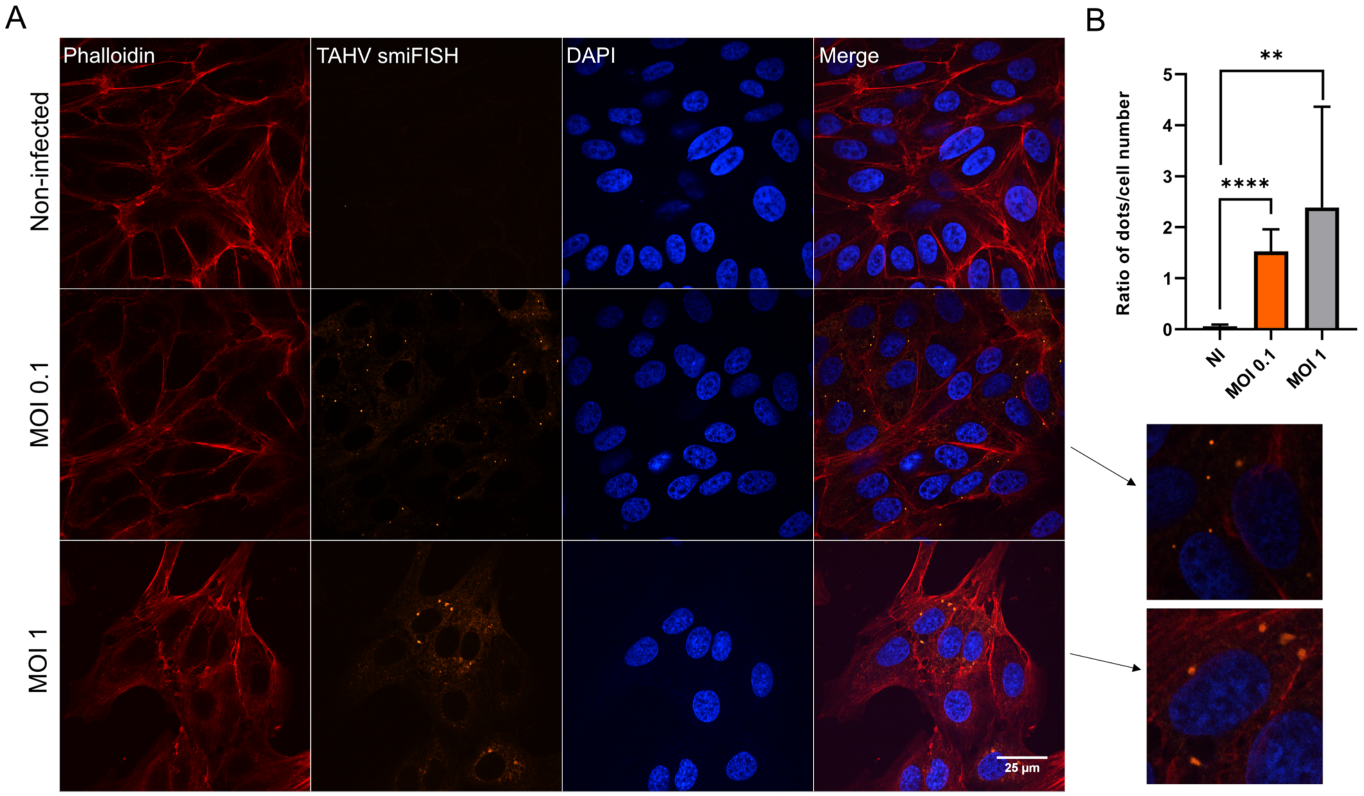

Figure EV4. Characterization of TAHV smFISH in Vero E6 cells.

(**A**) Vero E6 cells were infected with TAHV for 24 h at indicated MOI and stained with phalloidin (actin, red), TAHV smFISH (viral RNA, orange), and Dapi (nuclei, blue). Scale bar: 25 µm. Lower right panels correspond to two magnified crops from indicated conditions, highlighting dotted structures likely corresponding to viral factories. (**B**) Quantification of the number of large dotted structures from the TAHV smFISH staining in (**A**). Data are mean +/− SD from 10 fields of view per condition from an experiment. Unpaired t-test *p* value < 0.01 (**) or <0.0001 (****).
